# The rumen microbiome inhibits methane formation through dietary choline supplementation

**DOI:** 10.1038/s41598-021-01031-w

**Published:** 2021-11-05

**Authors:** Yang Li, Michael Kreuzer, Quentin Clayssen, Marc-Olivier Ebert, Hans-Joachim Ruscheweyh, Shinichi Sunagawa, Carmen Kunz, Graeme Attwood, Sergej Amelchanka, Melissa Terranova

**Affiliations:** 1 Institute of Agricultural Sciences, ETH Zurich, Universitaetstrasse 2, 8092 Zurich, Switzerland; 2 Institute of Microbiology, ETH Zurich, Vladimir-Prelog-Weg 4, 8093 Zurich, Switzerland; 3 Laboratory of Organic Chemistry, ETH Zurich, Vladimir-Prelog-Weg 3, 8093 Zurich, Switzerland; 4 AgResearch Ltd. Grasslands Research Centre, Palmerston North, 4442 New Zealand; 5 ETH Zurich, AgroVet-Strickhof, Eschikon 27, 8315 Lindau, Switzerland

**Keywords:** Computational biology and bioinformatics, Ecology, Microbiology, Molecular biology

## Abstract

Enteric fermentation from ruminants is a primary source of anthropogenic methane emission. This study aims to add another approach for methane mitigation by manipulation of the rumen microbiome. Effects of choline supplementation on methane formation were quantified in vitro using the Rumen Simulation Technique. Supplementing 200 mM of choline chloride or choline bicarbonate reduced methane emissions by 97–100% after 15 days. Associated with the reduction of methane formation, metabolomics analysis revealed high post-treatment concentrations of ethanol, which likely served as a major hydrogen sink. Metagenome sequencing showed that the methanogen community was almost entirely lost, and choline-utilizing bacteria that can produce either lactate, ethanol or formate as hydrogen sinks were enriched. The taxa most strongly associated with methane mitigation were *Megasphaera elsdenii* and *Denitrobacterium detoxificans*, both capable of consuming lactate, which is an intermediate product and hydrogen sink. Accordingly, choline metabolism promoted the capability of bacteria to utilize alternative hydrogen sinks leading to a decline of hydrogen as a substrate for methane formation. However, fermentation of fibre and total organic matter could not be fully maintained with choline supplementation, while amino acid deamination and ethanolamine catabolism produced excessive ammonia, which would reduce feed efficiency and adversely affect live animal performance.

## Introduction

Combating climate change caused by anthropogenic greenhouse gases is one of the most important challenges of our time. Methane (CH_4_) is a greenhouse gas that has a global warming potential 25 times that of CO_2_ in this respect^1^. Enteric fermentation accounts for 27% of total anthropogenic CH_4_ emission^2^. Therefore, mitigation of enteric CH_4_ is critical to limit emissions within the remaining CH_4_ budget^3^. Ruminants rely on a complex rumen microbiome consisting of bacteria, protozoa, fungi, archaea and viruses to digest feeds by enteric fermentation. Methanogenic archaea in the rumen, and less so in the hindgut, are the source of enteric CH_4_ from ruminants. The fermentation produces volatile fatty acids (VFAs), which are absorbed from the rumen and form a major source of metabolizable energy for the animal^4^. Apart from ammonia (NH_3_), the fermentation also produces CO_2_, gaseous hydrogen (H_2_) and methylated compounds as by-products. These by-products create a niche for the methanogenic archaea, which gain energy by using either dissolved H_2_ as sources of reducing potential needed for the reduction of CO_2_ or methylated compounds to CH_4_.

Physiologically, around 78% of the rumen archaea are hydrogenotrophic methanogens that reduce CO_2_ to CH_4_ by using dissolved H_2_ as a source of reducing potential according to the reaction CO_2_ + 4H_2_ → CH_4_ + 2H_2_O^5^. This includes members of the orders Methanobacteriales, Methanomicrobiales and Methanosarcinales. Approximately 22% of the rumen archaea are capable of using H_2_ to reduce methylated compounds, such as methanol to CH_4_ (CH_3_–OH + H_2_ → CH_4_ + H_2_O)^5^. This includes members of Methanobacteriales, Methanosarcinales and Methanomassiliicoccales (MMC). The Methanobacteriales are the most dominant order of rumen methanogens, with MMC being the second most dominant^5^. The MMC rely on a simplified methanogenesis pathway that utilizes methylated compounds, such as methylamines to generate energy, requiring only one mole of H_2_ per mole of CH_4_^6^. Therefore, they were predicted to have a lower threshold for dissolved H_2_ than other rumen methanogens^7^. As a consequence, they would be able to survive in situations of low H_2_ concentration which would be unfavourable for ATP production in other methanogens. Theoretically, methylotrophic methanogenesis would out-compete hydrogenotrophic methanogenesis at a low H_2_ concentration, as MMC would consume H_2_ and reduce dissolved H_2_ to a level that does not meet the thermodynamic conditions required to produce ATP by hydrogenotrophic methanogens^8^. Despite these thermodynamic and H_2_ threshold advantages, and temporary H_2_ limiting situations in certain periods of the feeding cycle, Methanobacteriales remains the dominant methanogen order in the rumen. Possible reasons for failure of MMC to dominate the rumen methanogen niche might be that MMC divert less energy towards ATP production from methanogenesis than other methanogens, or that their growth is limited by the insufficient availability of methylated substrates, such as mono-(MMA), di-(DMA) and trimethylamines (TMAs). The present study aimed to exploit this limitation and to use it to drive changes in the rumen microbiome towards a simplified rumen methanogen population with MMC as the dominant order by providing an abundant supply of either methylated substrate or their precursors as selection pressure. Such a simplified rumen methanogen population may be more vulnerable to CH_4_ mitigation strategies that have previously failed due to the high adaptability of a diverse methanogen population.

A preliminary experiment carried out to test the effects of different methylated compounds demonstrated that, while DMA and TMA significantly enhanced the MMC population, MMC did not outcompete other methanogens (Supplementary information and Fig. S1). Unexpectedly, it was found that the methylamine precursor choline^9^, caused a strong inhibition of CH_4_ production. Choline is a registered feed supplement and can be added to ruminant diets. It has been used before in a rumen-protected form to enhance milk yield, whereas choline undergoes extensive degradation within the rumen environment when provided in a non-protected form^10,11^. Consequently, a second aim of the present study was to investigate the underlying mechanism of CH_4_ mitigation imposed by choline. Experiments were carried out using the in vitro system Rumen Simulation Technique (Rusitec)^12^. To establish a dose–response relationship using choline and differences in efficiency between choline compounds, a dose–response and a main experiment were carried out. In the main experiment, in depth metagenomics and metabolomics approaches were applied for the identification of the choline-mediated mechanisms affecting the ruminal microbiome associated with methanogenesis the use of alternative H_2_ utilization pathways.

## Results

### Effect of methylated substrates on CH_4_ production and MMC abundance (preliminary experiment)

**Figure 1:**
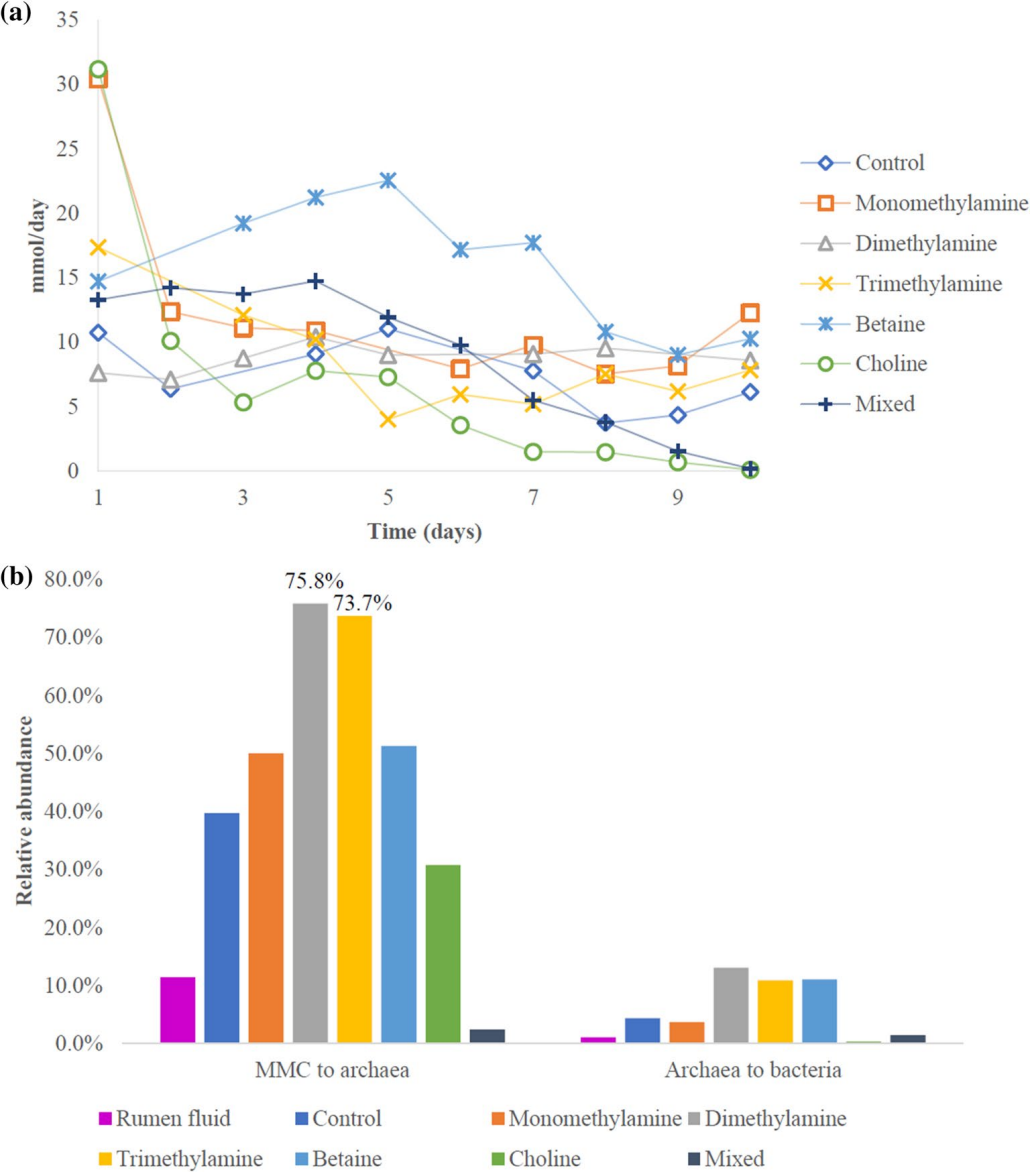
Effect of in vitro supplementation of mono-, di-, trimethylamine, betaine and choline at 200 mM and a mixed supplementation of all five at 100 mM each in Rusitec (preliminary experiment). (**a**) CH_4_ production per day over 10 days. (**b**) Relative abundance of MMC to archaea, archaea to bacteria at end of d10.

Supplementing of two of the three methylated substrates (MMA and TMA) and the TMA precursors betaine and ChCl numerically enhanced CH_4_ production initially compared to control, whereas this was different with DMA (Fig. 1a). However, ChCl and the mixed treatment caused a continuous decline from day 5 to near complete termination of CH4 production on day 10. The MMA and TMA treatments numerically reduced the relative abundance of MMC to archaea (Fig. 1b). Both treatments also numerically reduced the relative abundance of archaea to bacteria. All other methylated substrates numerically enhanced the relative abundance of MMC to archaea when provided alone. Although MMC was increased to above 70% of archaeal population by DMA and TMA, this was not sufficient to remove all other methanogens within the 10 days of supplementation.

### Response to choline dosage in CH_4_ production and NH_3_ concentration (dose–response experiment)

**Figure 2:**
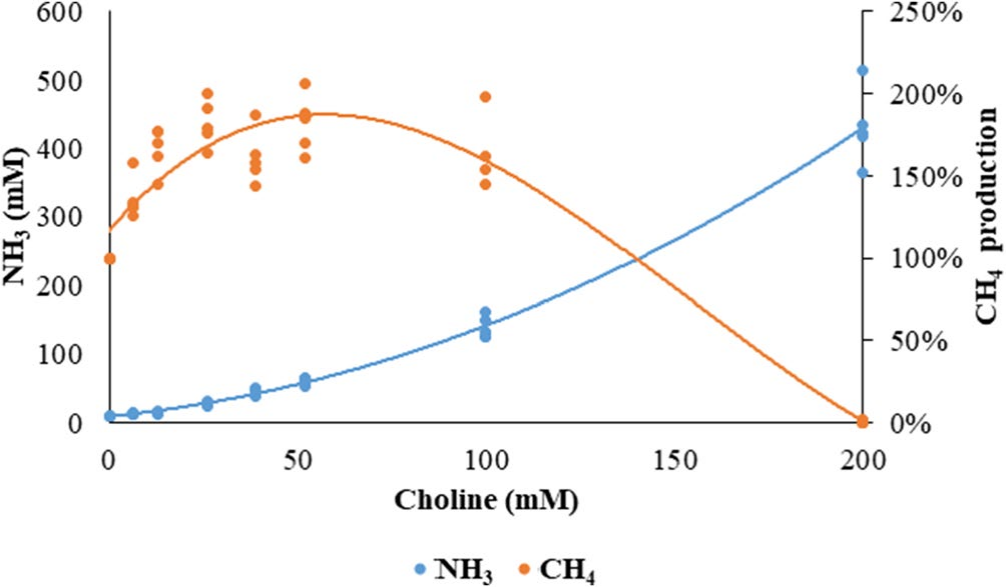
Dosage response of choline supplementation on CH_4_ and NH_3_ production. Choline was supplemented to artificial saliva at 0, 6.5, 13, 26, 39, 52, 100 and 200 mM for 15 days. The scatterplot displays the CH_4_ production in orange and NH_3_ concentrations in blue between day 11 and day 15, each dot representing measurement from a single day. The CH_4_ production was normalized to that of the 0 mM group. The CH_4_ production was fitted with a 3^rd^ order polynomial regression (y = 6 × 10^–07^ x^3^ − 0.0003 x^2^ + 0.0267 x + 1.1659, R^2^ = 0.908), the NH_3_ concentrations was fitted with a 2nd order polynomial regression (y = 0.0078 x^2^ + 0.5374 x + 9.1634, R^2^ = 0.982).

A supplementation of ChCl at 6.5, 13, 26, 39, 52, and 100 mM together with 10 μM CoM stimulated the CH_4_ production in a polynomial curve (Fig. 2). At 200 mM, the CH_4_ production was reduced to a level below the detection limit of the gas chromatograph. There was a large increase in the NH_3_ concentration of the incubation liquid when the ChCl dosage was increased.

### Effect of choline on methanogenesis and ruminal fermentation (main experiment)

**Figure 3:**
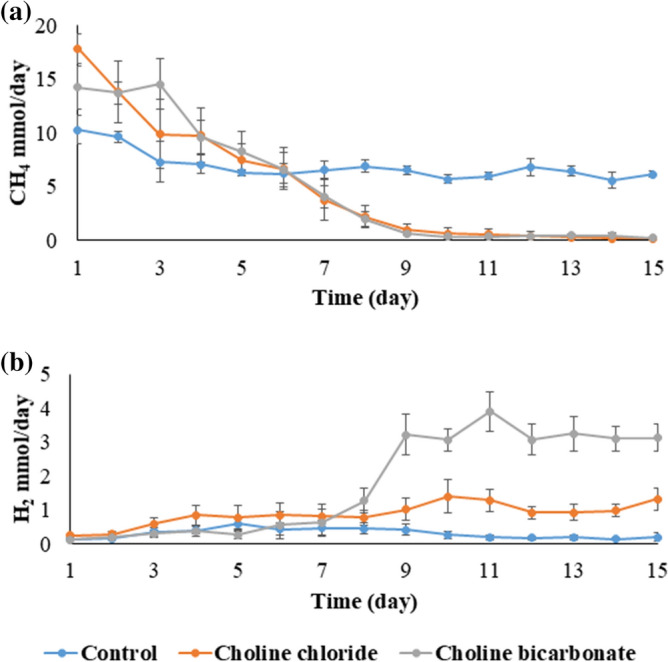
Effect of 200 mM of choline chloride and choline bicarbonate on (**a**) CH_4_ and (**b**) H_2_ production (n = 4). Average data obtained from day 11 to day 15 plotted with standard errors of the means as error bars.

A supplementation of ChCl at 200 mM initially tended (*P* < 0.10) to increase CH_4_ production compared to the control, but decreased it from day 6 onwards (Fig. 3a). On day 15, ChCl and ChHCO_3_ reduced the CH_4_ production to 2.1% and 3.5% of control respectively (*P* < 0.001). In three of the four replicates, there was no detectable CH_4_ production from day 12 onwards in ChCl treatment (Supplementary Table S1), while ChHCO_3_ was close but unable to reduce CH_4_ production to 0 mmol/day by the end of the experiment. Both ChCl and ChHCO_3_ increased the level of H_2_ accumulated by 7.1-fold and 16.9-fold of control, respectively (Fig. 3b and Supplementary Table S2). A fruity smell of the incubation liquid could be detected with both ChCl and ChHCO_3_ treatments, which may hint at production aromatic gas such as ethylene. The average pH of the incubation fluid differed (*P* < 0.001) between treatments from day 6 to day 15 (Table 1). The choline treatments also increased (*P* < 0.05) total VFA concentration, which was associated with an increased (*P* < 0.05) acetate proportion, whereas proportions of propionate and valerate decreased (*P* < 0.05). A reduction (*P* < 0.05) in both in vitro ruminal organic matter digestibility and neutral-detergent fibre digestibility was also observed. The NH_3_ concentration of the incubation liquid and the amount of N supplied and recovered in NH_3_ increased (*P* < 0.05) to about 30- and 20-fold higher than values found in control. From the methylated compounds detected in the incubation liquid on d15, only choline and TMA, but not MMA and DMA were substantially elevated (*P* < 0.05) by the choline treatments (Table 2). More than 90% of the choline was depleted, with the residual choline being lower by 70% with ChHCO_3_ than with ChCl. Ethanol was elevated by 192-fold (*P* < 0.05) and 153-fold (*P* < 0.05) of control in ChHCO_3_ and ChCl treatment respectively. The compounds with the strongest negative correlation to CH_4_ production were of TMA (r = − 0.99, *P* < 0.001), ethanol (r = − 0.95, *P* < 0.001) and NH_3_ (r = − 0.93, *P* < 0.001), while propionate concentration most strongly positively correlated with CH_4_ production (r = 0.86, *P* < 0.001). The variables most strong positive correlation with H_2_ production were concentrations of formate (r = 0.85, *P* < 0.001) and succinate (r = 0.84, *P* < 0.001).

**Table 1:** Effects of supplementation of 200 mM choline chloride and choline bicarbonate on incubation liquid traits (main experiment).

Supplement	Control^d^	Choline chloride	Choline bicarbonate	SEM	*P* value
pH	6.95^a^	6.31^b^	7.42^c^	0.070	< 0.001
NH_3_ (mmol/L)	20^a^	596^b^	580^b^	68.9	< 0.001
Total VFA (mmol/L)	68.0^a^	96.4^b^	84.6^ab^	5.32	0.044
**Molar proportions (% of VFA)**					
Acetate	43.8^a^	59.5^b^	67.6^b^	5.17	< 0.001
Propionate	20.3^a^	8.0^b^	4.9^c^	0.96	< 0.001
*n*-Butyrate	22.1	27.7	23.8	1.69	0.083
*Iso*-Butyrate	0.94	1.13	0.77	0.318	0.255
*n*-Valerate	7.73^a^	3.40^a^	2.70^b^	0.828	0.004
*iso*-Valerate	5.13^a^	0.39^b^	0.26^b^	0.215	< 0.001
Bacteria (× 10^8^/mL)	4.88	5.30	5.93	0.654	0.331
Protozoa (× 10^4^/mL)	1.27	1.02	1.00	0.149	0.270
**Nutrient disappearance (g/g supply)**					
Organic matter	0.738^a^	0.662^b^	0.645^b^	0.0117	< 0.001
Neutral detergent fibre	0.533^a^	0.407^b^	0.387^b^	0.0097	< 0.001
**N turnover (mg/day)**					
N supply (basal diet + choline)	423	1551	1551	‒	‒
N recovered in NH_3_	115^a^	3363^b^	3272^b^	388.9	< 0.001

Averages of days 6 to 15; mean values with highest standard error of the means (SEM); n = 4). VFA: volatile fatty acids.

^a–c^Mean values within a row without common superscripts are significantly different (*P* < 0.05).

^d^Basal diet only.

**Table 2:** Effects of supplemention of 200 mM of choline chloride and choline bicarbonate on incubation liquid metabolites as measured by proton nuclear magnetic resonance (main experiment).

Metabolite (mM)	Inoculum	Control	Choline chloride	Choline bicarbonate	SEM	*P* value
Choline	0.07^a^	0.06^a^	18.44^b^	5.45^a^	6.836	< 0.001
Monomethylamine	0.113	0.035	0.116	0.037	0.0572	0.220
Dimethylamine	0.025^ab^	0.004^a^	0.105^b^	0.030^ab^	0.0359	0.0234
Trimethylamine	0.2^a^	0.1^a^	129.8^b^	134.7^b^	5.34	< 0.001
Ethanol	0.41^a^	0.34^a^	51.92^b^	65.51^c^	5.514	< 0.001
Acetaldehyde	0	0.008	0.192	0.287	0.2483	0.351
Glycerol	5.40	5.71	7.52	9.17	3.711	0.813
Lactate	0.136	0.133	0.271	1.320	0.8313	0.185
Succinate	0.066^a^	0.042^a^	0.043^a^	1.531^b^	0.2649	< 0.001
Formate	0.038^a^	0.036^a^	0.125^a^	2.491^b^	0.5442	< 0.001
Methanol	0.021^a^	0.016^a^	1.392^b^	0.676^ab^	0.3149	< 0.001
Phenylpropionate	0.565^a^	0.317^b^	0.200^b^	0.195^b^	0.0384	< 0.001
2-Methyl-butyrate	0.689^a^	3.132^b^	1.060^a^	0.833^a^	0.6264	0.0012

All groups other than inoculum (i.e., day 0) are day 15 measurements; mean values with highest standard error of the means (SEM); n = 4.

^a–c^Mean values within a row without common superscripts are significantly different (*P* < 0.05).

### Effect of choline and its chemical form on the rumen microbiome (main experiment)

**Figure 4:**
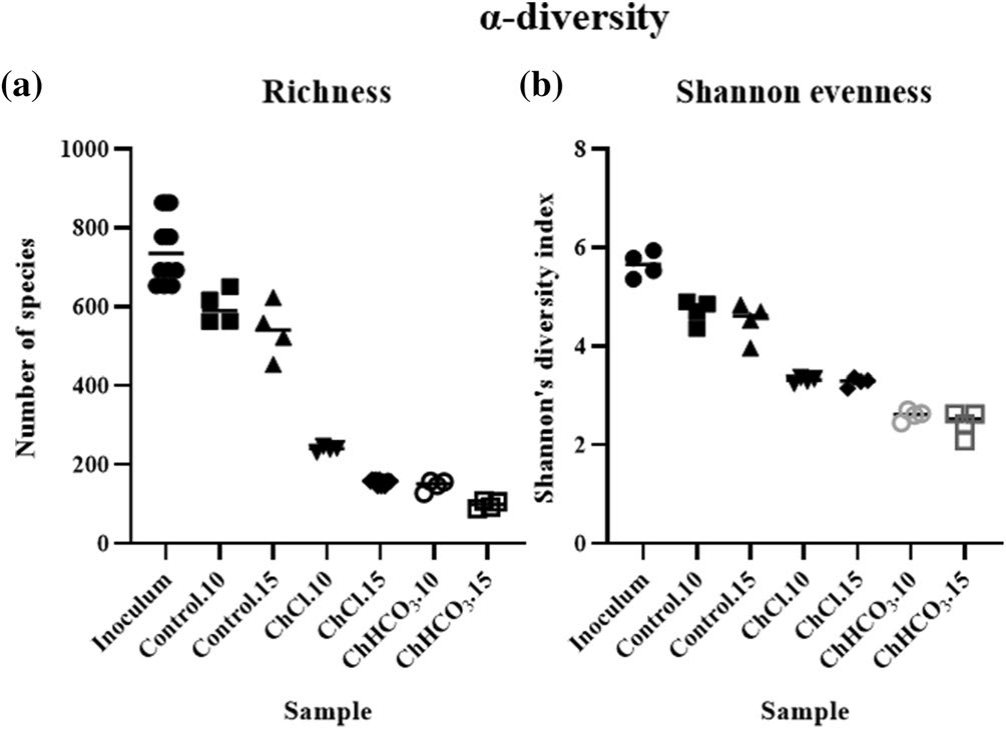
α-Diversity as assessed by (**a**) species richness and (**b**) Shannon evenness of inoculum, control, choline chloride (ChCl) and choline bicarbonate (ChHCO_3_) (day 10 (.10) and 15 (.15) in scatter plot) (data from main experiment).

**Figure 5:**
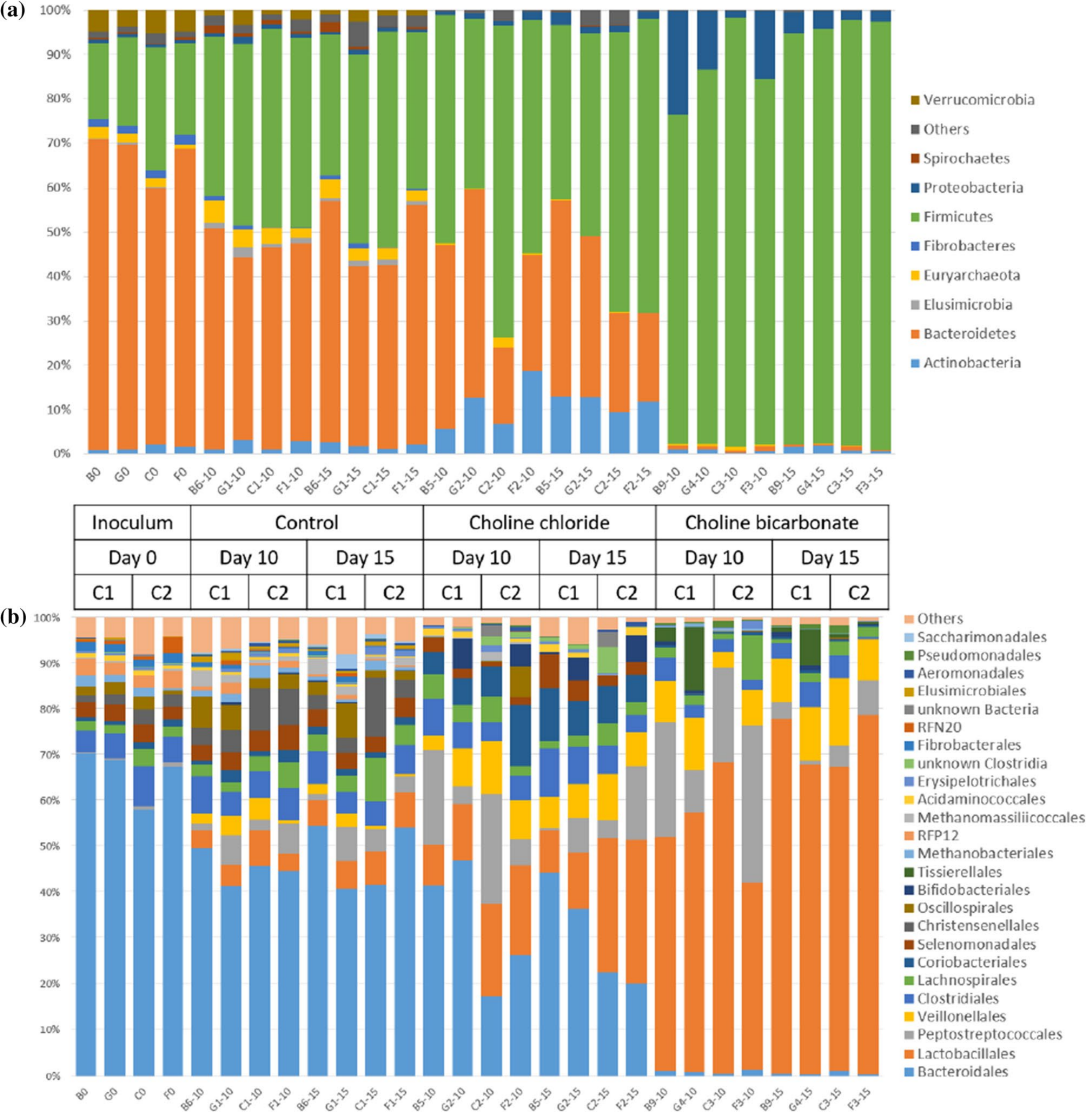
Stacked column graph depicting the relative abundances and distribution of (**a**) the nine phyla with ≥ 1% abundance in one or more samples comprising 98.6% of all taxa and (**b**) the 25 most highly abundant orders comprising 94.4% of all taxa. The remaining phyla and orders, respectively, were pooled as ‘Others’. C1: Cow 1, C2: Cow 2. Relative abundances were obtained through mOTUs2 profiler (v2.5) (data from main experiment).

The effects of two forms of choline supplementation on total bacteria and total protozoa counts were not significant (Table 1). This was different concerning the composition of the microbiome. The changes in α-diversity showed that, compared to the species richness of the rumen fluid used for inoculation, 15 days of treatment with ChCl and ChHCO_3_ reduced species number to 21.2 ± 1.6% (mean ± standard error) and 13.4 ± 1.4% respectively, while under control condition 73.8 ± 8.7% of the species could be maintained (Fig. 4a). The Shannon evenness was also altered from 5.65 ± 0.13 (inoculum) to 3.28 ± 0.04 (ChCl), 2.44 ± 0.13 (ChHCO_3_) and 4.50 ± 0.19 (control) on d15 (Fig. 4b). The β-diversity also illustrated a difference between treatment groups and control (Supplementary Fig. S1). The permutation analysis of variance indicated that microbiome composition differs between groups (*P* < 0.001). The relative prokaryotic abundance is illustrated in Fig. 5. At the phylum level (Fig. 4a), the ChCl treatment increased the relative abundance of Actinobacteria, while the ChHCO_3_ treated microbiome was dominated by Firmicutes, along with an increased proportion of Proteobacteria. A marked reduction of Euryarchaeota was observed in both treatments indicating a decline of the methanogenic archaea. At the order level, the control group showed an increased relative abundance of Lactobacillales, Peptostreptococcales and Veillonellales. The ChCl treated microbiome had an even higher relative abundance of the same orders of microbes and of Coriobacteriales. By contrast, the ChHCO_3_-treated microbiome was dominated by Lactobacillales. The relative abundance overview at species level for all taxa with 1% abundance or more in one or more sample is listed in Supplementary Table S3.

The ChCl treatment increased the abundance of *Olsenella umbonata*, Anaerovoracaceae spp. and unknown *Olsenella*_B (Supplementary Table S4 and S5). The ChHCO_3_ treatment increased the abundance of *Enterococcus avium*, *Enterococcus gallinarum*, *Alkaliphilus* spp and *Globicatella sanguinis* (Supplementary Table S6 and S7). Both choline treatment groups were additionally pooled in order to identify conservation of differentially abundant species that may have contributed to CH_4_ mitigation on d15. This comparison identified 227 less abundant species, including five clusters of *Methanobrevibacter* and two clusters of MMCs; eight species were identified to be more abundant (Supplementary Table S8). In addition, eight mOTUs clusters were identified by R_s_ to be negatively associated with CH_4_ production and 15 mOTUs clusters were positively associated with H_2_ production (Table 3). Among the species associated with CH_4_ mitigation, *M. elsdenii* (6.24% average relative abundance) possessed the strongest association, *M. elsdenii* also correlates with NH_3_ (r = 0.801, *P* < 0.001). *Enterococcus gallinarum* (4.06% average relative abundance) was most strongly associated with H_2_ production. Seven mOTUs were positively correlated with ethanol concentration in the incubation liquid, and six of them were negatively correlated with CH_4_ production.

**Table 3:** Metagenomic-based Operational Taxonomic Units (mOTUs) associated with CH_4_ mitigation, H_2_ production and ethanol concentration as identified by Spearman’s Rank correlation coefficient (Rs) (data from main experiment).

Taxonomy	mOTU^a^	Spearman R_s_	*P*-value	BH *p*adj	% average abundance
**mOTUs negatively associated with CH**_**4**_ **production**					
*Megasphaera elsdenii*	ref_mOTU_v25_01516	− 0.743	< 0.001	< 0.001	6.246
*Denitrobacterium detoxificans*	ref_mOTU_v25_06442	− 0.683	< 0.001	0.00205	0.098
*Denitrobacterium detoxificans*	rumen_mOTU_2272	− 0.674	< 0.001	0.00235	0.078
unknown *Lachnospiraceae*	rumen_mOTU_727	− 0.640	< 0.001	0.00456	0.239
*Lachnospira multipara/pectinoschiza*	ref_mOTU_v25_03833	− 0.617	0.00133	0.00695	0.938
*Streptococcus equinus*	ref_mOTU_v25_00901	− 0.540	0.00651	0.024	5.877
*Lactobacillus ruminis*	ref_mOTU_v25_01239	− 0.535	0.00711	0.026	1.608
*Streptococcus sp.*	ref_mOTU_v25_00902	− 0.487	0.016	0.046	0.107
**mOTUs positively associated with H**_**2**_ **production**					
*Enterococcus gallinarum/saccharolyticus*	ref_mOTU_v25_03214	0.751	< 0.001	0.020	4.062
*Streptococcus sp.*	ref_mOTU_v25_00902	0.740	< 0.001	0.020	0.107
*Streptococcus equinus*	ref_mOTU_v25_00901	0.716	< 0.001	0.020	5.877
*Enterococcus avium*	ref_mOTU_v25_02620	0.706	< 0.001	0.020	6.564
*Pseudomonas mendocina*	ref_mOTU_v25_00237	0.700	< 0.001	0.020	0.008
*Clostridium botulinum/sporogenes*	ref_mOTU_v25_01616	0.699	< 0.001	0.020	0.003
*Enterococcus sp.*	ref_mOTU_v25_02783	0.688	< 0.001	0.020	0.575
*Pseudomonas sp.*	ref_mOTU_v25_00235	0.671	< 0.001	0.022	0.029
*Pseudomonas guguanensis/mendocina*	ref_mOTU_v25_00238	0.640	< 0.001	0.026	0.211
unknown *Alkaliphilus*	rumen_mOTU_765	0.598	0.00202	0.039	3.506
*Proteobacteria sp*.	ref_mOTU_v25_00095	0.580	0.00299	0.043	0.003
unknown *Clostridiales*	rumen_mOTU_24	0.568	0.00378	0.048	0.029
unknown *Clostridium_J*	rumen_mOTU_2237	0.565	0.004	0.049	0.023
*Streptococcus sp.*	ref_mOTU_v25_00900	0.565	0.00401	0.049	0.015
unknown *Methanobrevibacter*	rumen_mOTU_404	0.563	0.00418	0.049	0.015
**mOTUs positively associated with ethanol concentration**					
*Megasphaera elsdenii*	ref_mOTU_v25_01516	0.949	< 0.001	< 0.001	5.047
*Lachnospira multipara/pectinoschiza*	ref_mOTU_v25_03833	0.812	< 0.001	0.016	0.665
*Denitrobacterium detoxificans*	rumen_mOTU_2272	0.784	< 0.001	0.031	0.410
*Denitrobacterium detoxificans*	ref_mOTU_v25_06442	0.778	< 0.001	0.031	0.000
*unknown Lachnospiraceae*	rumen_mOTU_727	0.773	< 0.001	0.032	0.197
*Selenomonas ruminantium*	ref_mOTU_v25_04318	0.755	< 0.001	0.042	0.024
*Streptococcus sp.*	ref_mOTU_v25_00902	0.746	< 0.001	0.046	0.065

Spearman R_s_: Spearman rank correlation coefficient. BH *padj*: Benjamin Hochberg false discovery rate adjusted *p*-value. Inoculum was excluded from this analysis (n = 24) as no gas production measurements were available. In case of ethanol concentration, this analysis only includes inoculum and day 15 samples with corresponding hNMR metabolite data (n = 16). Only mOTU clusters that meet the required *P* < 0.05 and Benjamini–Hochberg *padj* < 0.05 cutoff are presented. ^a^Unique mOTU ID within mOTUs database (https://motu-tool.org/).

## Discussion

Choline has a marked effect on methanogenesis. The results of the preliminary experiment indicated that, although some methyl compounds did enrich MMC, this did not allow MMC to out-compete other methanogens to the point where they were the only methanogen remaining. In fact, the study showed that choline, after at first (day 1) promoting the rumen methanogen population and CH_4_ formation at high supplementation level, eventually led to a near complete cessation of the methanogenic activity. Methanogens were negatively affected already from day 2 onwards, as shown by the decline of CH_4_ production. In order to ensure that the influence on CH_4_ was due to choline itself, two different chemical forms of choline –ChCl and ChHCO_3_—were supplemented. The maximal level of CH_4_ reduction achieved at 200 mM was nearly the same with ChCl (98%) and ChHCO_3_ (97%).

**Figure 6:**
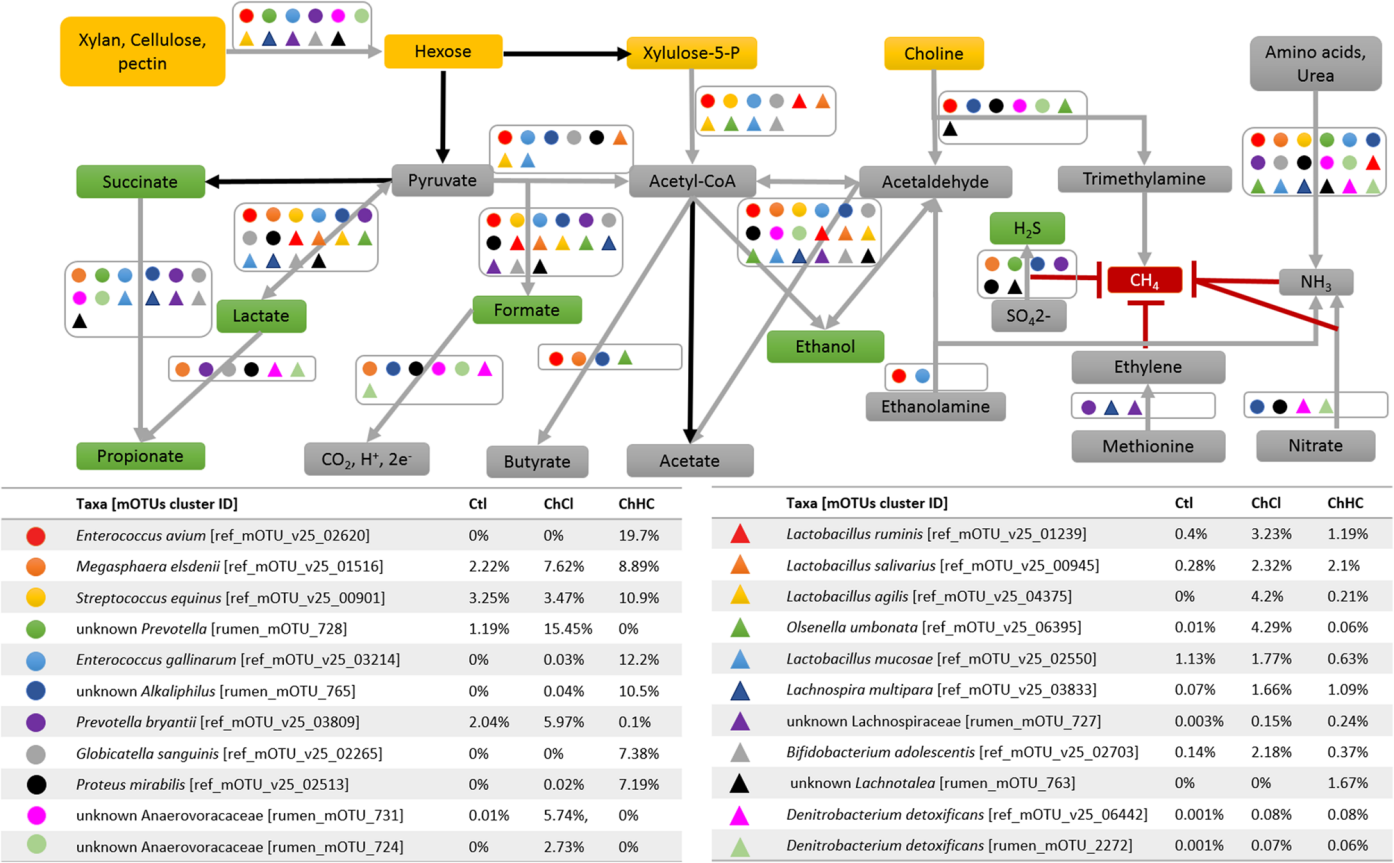
Proposed metabolic pathway of VFA production and CH_4_ mitigation by abundant species and species of interest. The yellow boxes represent nutrients from feed bags and supplements supplied to Rusitec, the green boxes represent alternative H sinks, the red box represents CH_4_, the other metabolites are shown in grey boxes. Black arrows represent pathways shared by all species, grey arrows are pathway only the species depicted by the legend are known or predicted to possess. The legend representing each species is shown along with their average relative abundance from day 10 and day 15 in each treatment group.

The present study henceforth sought to answer how these phenomena can be explained using results from ruminal fermentation and rumen microbiome composition. According to the results of the hNMR analysis, more than 90% of the choline was utilized (Table 2) and likely converted to TMA and acetaldehyde by choline TMA-lyase^13^. The substantial increase found in TMA concentration in the incubation with both forms of choline along with CH_4_ mitigation might therefore be an indicator of inhibition of methanogenesis. The supplementation of 200 mM TMA did not reduce CH_4_ production. Therefore, it is likely that the acetaldehyde as end product of choline metabolism may play an active role leading to the CH_4_ mitigation, and choline may act as a selection pressure to encourage the bacteria that can take advantage of it. The group of bacteria possessing choline TMA-lyase would be the first to benefit from acetaldehyde. The choline TMA lyase and its activating enzyme have been identified in the differentially abundant mOTUs clusters, including members of the Anaerovoraceae and *Olsenella umbonata* enriched by ChCl supplementation and *Enterococcus avium*, *Alkaliphilus* spp., *Proteus mirabilis/vulgaris* and unknown *Lachnotalea* spp. enriched by ChHCO_3_. Accordingly, the two forms of choline stimulated entirely different species capable of degrading choline^13^. *E. avium*, *Proteus mirabilis*, unknown *Alkaliphilus* and unknown *Lachnotalea* could be the species metabolizing choline in the ChHCO_3_ group, and *Olsenella umbonata* and unknown Anaerovoraceae those metabolizing choline in the ChCl group (Fig. 6). All but *Lachnotalea* possess *eut* gene clusters associated with choline utilization via microcompartment^13,14^. Inside microcompartments choline can be metabolized to acetaldehyde and ammonia, the acetaldehyde may subsequently be converted to ethanol and acetate^15^. All of the aforementioned species can produce acetate, but only a subset can produce propionate and butyrate (Table S11). The reductive acetogen *Alkaliphilus* may have contributed to the elevated acetate concentration in the treatment groups. The two *Enterococcus* species dominant with the ChHCO_3_ treatment are also predicted to be able to metabolize ethanolamine. The organisms that can cleave choline to TMA and acetaldehyde are presented in Fig. 6 along with the average abundance of each species in each condition. Acetaldehyde enters the central carbon metabolism and thus can lead to the production of lactate, succinate, ethanol and formate, which are alternative H_2_-sinks, and influence the downstream microbial crosstalk. Figure 6 illustrates simplified pathways of ruminal degradation of choline and of certain structural carbohydrates (xylan, cellulose, pectin) as well as of the production of ethanol, formate, succinate and VFA along with the predicted capability of species of high abundance and species of interest.

Choline affects ruminal NH_3_ formation, which can potentially inhibit CH_4_ formation. Theoretically even the very high level of 200 mM choline chloride could be used in livestock nutrition in the European Union, because the responsible organization, the European Food Safety Authority^16^, has not set a limit for this particular supplement in feed in their regulations. However, the NH_3_ concentration in the incubation liquid found at 200 mM choline supplementation by far exceeded the critical level for NH_3_ toxicity of > 110 mM^17^. The increase in NH_3_ is likely to come from choline metabolism, but the total amount of NH_3_-N produced exceeds that added as choline-N, so NH_3_ is likely also produced from sources other than choline.

The high level of NH_3_ produced by choline treatment may also contribute to the lowering of CH_4_ production. At the physiological ruminal pH of 6.5 or lower, almost all NH_3_ exists in the form of the NH_4_^+^ ion^18^. NH_3_ can pass through the cell membrane and requires cellular H^+^ to form NH_4_^+^. In methanogens this may divert H^+^ away from methanogenesis^19^. When methanogen cultures were inhibited with 400 mM NH_4_Cl, the cytoplasmic NH_3_ concentration ranged from 100 mM to above 200 mM^19^. The ammonia concentration in the choline supplemented Rusitec incubation liquid was well above 400 mM in the current study, which suggests that NH_3_ may be acting to cause CH_4_ inhibition. Signs of toxicity to the ruminant were observed when ruminal NH_3_ exceed 110 mM^20^. The high level of NH_3_ registered in the present experiment therefore suggests this treatment should never be carried out in live animals.

In a previous study^21^, there was increased production of N containing microbial compounds, likely due to the improvement in efficiency of synthesis of such compounds when methanogenesis was inhibited, despite decreased OM digestibility. Also, some species identified in the present study are able to ferment amino acids and produce NH_3_ from them, while some are able to reduce nitrate to NH_3_, or catabolize ethanolamine to produce NH_3_^22^. A particularly important role in explaining the NH_3_ excess in the present study could be attributed to the presence of *M. elsdenii* (ref_mOTU_v25_01516), its high abundance correlates positively with NH_3_ concentration (r = 0.801, *P* < 0.001). This species has been observed to produce NH_3_ nearly as fast as obligatory amino acid fermenting bacteria^23^. In the present study, the N retained by NH_3_ exceeded that of the N input from the feeds provided with the nylon bags, which suggest microbial N fixation may have occurred from the N_2_ gas used to keep Rusitec anaerobic^24^. This phenomenon has been described previously^25^. It is possible that the inhibition of methanogenesis may increase nitrogenase activity, as was found under rice paddy conditions^26^. However, nitrogenase requires 16 mol of ATP to fix one mole of N_2_^27^, which makes it inferior in a competitive environment such as the rumen. It is possible to power the nitrogenase by a proton membrane potential via a FixABC membrane complex^28^, but this operon was only predicted to be present in *Proteus mirabilis* which does not harbour a nitrogenase reductase complex. Instead, nitrogenase reductase has been identified in *Prevotella bryantii* and *Lachnospira multipara*^29^, and predicted in *M. elsdenii*, i.e., members of the Lachnospiraceae, *Lachnotalea* spp. and Anaerovoracaceae spp. Therefore, it is unlikely the N fixation could be channelled by FixABC and the plausibility of N fixation with high choline supplementation requires further study.

Ethanol was among the metabolites most strongly associated with reduced CH_4_ production. Its high concentration in the incubation liquid suggests that this compound is an important alternative H_2_-sink^30^, which could have been a consequence from microbiome adaptation to the absence of methanogenesis. Unlike other alternative H_2_-sinks such as succinate and lactate that are readily converted to propionate by bacteria, ethanol might have been primarily utilized by the methanogens^31,32^. Therefore, the lack of methanogens likely led to the accumulation of ethanol.

Alternative electron acceptors, for example sulphate (SO_4_^2−^ + 4H_2_ + H^+^ → HS^-^ + 4H_2_O, ∆G = − 234 kJ), nitrate (NO_3_^-^ + H_2_ → NO_2_^-^ + H_2_O, ∆G = − 161 kJ) and nitrite (NO_2_^-^ + 3 H_2_ + 2 H^+^ → NH_4_^+^  + 2H_2_O, ∆G = − 519 kJ) are thermodynamically more favourable than methanogenesis (CO_2_ + 4 H_2_ ⟶ CH_4_ + 2 H_2_O, ∆G = − 134 kJ)^33^. This makes sulphate-reducing bacteria (SRB) and nitrate-reducing bacteria (NRB) effective in H competition with methanogens, when sufficient substrate is present^34,35^. Both SRB and NRB were detected in the choline supplemented treatments (Fig. 6). Predicted SRB include *M. elsdenii*, *Prevotella* spp*.*, *Alkaliphilus* spp., *Prevotella bryantii*, *Proteus mirabilis*^36^ and *Lachnotalea* spp. A high concentration of H_2_S is associated with polioencephalomalacia and is detrimental to the ruminant^37^. Predicted NRB include *Alkaliphilus* spp., *Proteus mirabilis* and *Denitrobacterium detoxificans*^38^. Among these organisms, *M. eldsenii* and *D. detoxificans* were negatively correlated with CH_4_ production in the present experiment.

Furthermore, *D. detoxificans* gains energy by oxidizing nitrogenous compounds such as nitroethane, 2-nitroalcohol and 3-nitro-1-propionate, using trimethylamine from choline metabolism as electron acceptor^38^. These nitrogenous compounds can be accumulated in forages, in particular legumes such as the alfalfa used in the basal diet in the present study and be made available within the rumen^39^. The N compounds mentioned are known to act as methanogen inhibitors^40^, and thus may have contributed to the CH_4_ inhibition observed in the present study.

Although lactate and succinate were not detected in high concentrations in the incubation liquid of the current study, the high abundance of lactate producing lactic acid bacteria (LAB), which led the lactate consuming *M. elsdenii* to prosper. *M. elsdenii* is negatively correlated to CH_4_ production, which suggest strongly that lactate were a prominent H_2_-sink^41^ in the present study as well. Both lactate and succinate are intermediate metabolites that can be readily converted to propionate^42^ and the production of propionate could compete with CH_4_ production^43^. However, a significant decrease of propionate was observed in the choline treatments. In previous batch cultures, inhibition of CH_4_ was accompanied by metabolic H redirected from acetate to propionate; however, continuous cultures like Rusitec behave differently: when CH_4_ was mitigated by > 50%, generally no overall metabolic H redirection to propionate or butyrate had been observed^30,44^. It was speculated^29^ that the H was diverted to other H_2_ sinks or microbial cell mass. This means that lactate may not have been primarily converted to propionate in the present study. Lactate can also be oxidized to pyruvate by an NAD-independent lactate dehydrogenase^45^ connected to electron bifurcation from electron-transferring flavoprotein Etf, butyryl-CoA dehydrogenase Bcd and ferredoxin/flavodoxin-NAD^+^ reductase Rnf complex^46,47^. All of these enzymes are present in *M. elsdenii*, which may then use the pyruvate to increase microbial cell mass^41^. The increased abundance of LAB and radical decline of Bacteroidetes in the ChHCO_3_ treatment is similar to what has been observed during rumen acidosis, where the pH sensitive Gram negative Bacteroidetes perish^48^. However, instead of acidosis, the pH increased in the ChHCO_3_ treatment due to the buffering capacity of bicarbonate, which suggests that there is more than pH that caused the Bacteroidetes to perish, perhaps an inhibition by LAB^49^.

The production of CH_4_ from H_2_ by methanogens prevents H_2_ accumulation and thereby avoids inhibition of fermentation of nutrients via negative feedback loops, especially of fibre where most H_2_ is produced. Therefore, the inhibition of CH_4_ production by choline metabolism was expected to have a negative effect on ruminal nutrient degradation as observed earlier in continuous culture experiments^30^. Hydrolysis of plant structural carbohydrates xylan, cellulose and pectin releases hexoses, which are metabolized via the glycolytic pathways to produces pyruvate, a branching point to acetate, propionate, butyrate, lactate, formate or ethanol production^44^. The individual steps in the glycolytic pathway are not affected by the increased H_2_ partial pressure, but the regeneration of NAD^+^ required for glycolysis is negatively impacted^50^, and organisms may be driven to use alternative H_2_-incorporating reactions, such as succinate, lactate and ethanol production which directly regenerates NAD^+^. In this way, the microbiome is able to adapt, and the surviving microbiome likely harbours alternative H_2_-incorporating pathways such as lactate or succinate-mediated propionate production^32,47^. This allows fermentation to continue but at a reduced capacity^51^.

Assuming the H_2_ concentration between liquid phase and gas phase is in equilibrium according to Henry’s law, both ChCl and ChHCO_3_ in fact increased H_2_ partial pressure (7.1-fold and 16.9-fold H_2_ accumulation compared to control, respectively), which governs the Gibbs free energy (∆G) of VFA production^43^. Furthermore, the ∆G must be greater than the minimum amount of energy required for ATP production for the reaction to be viable in bacteria. Therefore, the surviving microbiome after choline treatment is likely capable of decoupling energy production from H_2_ partial pressure by various means, including usage of alternative H_2_-sinks.

The richness of the microbiome in the control group after 15 days of operation indicates that Rusitec is a good simulation system for the rumen prokaryotes. The microbiome revealed a decline of Euryarchaeota, i.e., the methanogens, in the choline treatment groups, which corresponds to the reduced CH_4_ formation. Some methanogens have syntrophic interaction with specific H_2_ producers via adhesins^52^. The reduced alpha diversity suggests that syntrophic interaction may have been broken, which could contribute to the reduced CH_4_ production. All of the most differentially abundant taxa found in the present study are either able to utilize H_2_ and produce metabolites such as lactate and ethanol, or they are able to make use of the alternate H_2_-sink metabolites produced by other bacteria. It is unknown whether the use of alternative H_2_ sinks is the result of CH_4_ reduction, or a contributor to CH_4_ reduction.

## Conclusion

As a model, treatment with choline, especially in the form of choline chloride, has demonstrated a new way to inhibit methanogenesis and to reduce CH_4_ to below the detection limit for in vitro continuous culture systems. This treatment could be used to study how the energy, otherwise lost through CH_4_ production could be redirected, and how rumen fermentation takes place in the absence of methanogenesis. This treatment reduces digestibility and massively enhances ruminal ammonia concentration, and is thus not suitable to be carried out in live animals for animal health and welfare reasons.

## Methods

### Experimental design

All methods were performed in accordance with the relevant guidelines and regulations.

The preliminary experiment was designed to investigate which methyl-compound enhanced the MMC community most strongly. Different methylated substrates, namely MMA, DMA and TMA, as well as the TMA precursors betaine and choline (provided as chloride (ChCl)), all from Sigma-Aldrich (St. Louis, MO, USA), were supplemented at 200 mM (equivalent to 750 g/kg basal diet supplied at 15 g dry matter/day) to the artificial saliva flowing into the Rusitec. In addition, an aliquot of an equimolar mixture of all five supplements was included at 100 mM each. The duration of the experiment was 10 days.

In the dosage response experiment, the dosage of ChCl required to achieve CH_4_ reduction was investigated during 15 days in a dose–response design within one Rusitec run. The ChCl was supplemented via artificial saliva at 0 (control), 6.5, 13, 26, 39, 52, 100, and 200 mM, by continuous flow into the Rusitec.

In the main experiment, three treatments were investigated in four 15-day Rusitec runs in a complete, randomized design. Treatment 1, control: 0 mM ChCl, Treatment 2: 200 mM ChCl, Treatment 3: 200 mM choline bicarbonate (ChHCO_3_) were supplemented with the artificial saliva in four replicates each. In all experiments, artificial saliva was supplemented with 10 µM coenzyme M (CoM; Sigma-Aldrich), as MMC relies on external supply of CoM to grow^6^ and a deficiency thereof might have effects on methanogenesis not related to the supply with methylated substrate.

### Origin of the rumen fluid

The starting rumen fluid was collected from two available lactating rumen-cannulated Brown Swiss cows. Cow 1 was fed ryegrass hay ad libitum and concentrate (1 kg/day), cow 2 was fed hay from a biodiverse meadow ad libitum. The rumen fluid was always collected at 07:00 a.m. just prior to refilling hay troughs and offering concentrate (cow 1 only). Procedures imposed on the rumen-fluid donor animals in the present study were approved by the Committee on Animal Experimentation (Ethics Committee) of the Cantonal Veterinary Offices of Zurich (Licence no. ZH 38/14; cow 1) and Berne (Licence no. VB BE 20/17; cow 2).The rumen fluid from each cow was separately used as starting inoculum in two of the four runs each in the main experiment to be able to offer two biological replicates each. Rumen fluid from cow 1 was used for both preliminary experiments. The rumen fluid was kept warm in a thermos flask during transport and inoculation took place within 2 h after rumen fluid harvest. The rumen fluid was strained through four layers of medical gauze (pore size 1 mm) prior to transfer into the Rusitec vessels.

### Operation of the Rusitec

An 8-fermenter Rusitec, as described in detail by Soliva and Hess^12^, was used for all experiments. The incubation was initiated with a mixture of 700 mL strained rumen fluid and 200 mL of pre-warmed artificial saliva added to each 1 L fermenter. Temperature was maintained at 39.5 °C with the help of a heated water bath. A basal diet consisting of 15 g dry matter/day of ryegrass hay, wheat flakes and soybean meal (1:0.7:0.3) was provided in all experiments in nylon bags with a pore size of 100 µm. Incubation of the bags lasted for 2 days each. This was accomplished by two nylon bags where on the first day one of them contained about 40 g fresh matter of solid ruminal contents. In addition, 75 mg/day of a vitamin-mineral mixture was added to the basal diet. This mixture contained, per g, Ca, 140 mg; P, 70 mg; Na, 80 mg; Mg, 30 mg; Se, 0·015 mg; vitamin A, 150 mg; vitamin D_3_, 3 mg; vitamin E, 2·5 mg, following Soliva et al*.*^53^. The artificial saliva^12^ had a composition ensuring a continuous supply of substrates required for optimal fermentation. The artificial saliva was sterilized by passing a 0.2 μm filter and stored in 10 L Nalgene autoclavable carboy (Thermo Fisher Scientific, Waltham, MA, USA). All Tygon tubes connecting the artificial saliva to the fermenter were also sterilized prior to the experiment. The overflown incubation liquid was collected in flasks to measure flow rate and immediately frozen at − 20 °C to terminate fermentation. To simulate the rumen washout effect, the average artificial saliva flow rate was 403 mL/day, equivalent to a dilution rate of 40.3%/day.

### Sample collection

Incubation liquid samples were taken daily 3 h prior to feed bag exchange to assess pH, NH_3_ concentration and VFA content. A portion of the incubation liquid was centrifuged for 5 min at 4000*g*, the supernatant was stored at − 20 °C for later high performance liquid chromatography (HPLC) and proton nuclear magnetic resonance (hNMR) analysis. After 48 h of incubation, respectively, the feed bags were processed for subsequent nutrient analysis as described by Soliva et al.^53^, detergent fibre fractions were assessed by Fibertherm system FT 12 (Gerhardt GmbH & Co. KG, Koenigswinter, Germany) as described by Terranova et al.^54^. The fermentation gases were collected during 24 h periods in gas-tight aluminium bags (TECOBAG 8 L, PETP/AL/PE-12/12/75 quality; Tesserau Container, Bürstadt, Germany). A portion of the initial rumen fluid inoculum and the subsequent incubation liquid were snap frozen in liquid nitrogen and stored at − 80 °C for microbiome assessment via metagenomics.

### Incubation liquid and fermentation gas analysis

Protozoal and bacterial counts in the incubation liquid were obtained daily with Neubauer haemocytometers (0.1 and 0.02 mm depth, respectively; Blau-Brand, Wertheim, Germany) following the manufacturer’s recommendation. The pH and NH_3_ concentration were measured by corresponding electrodes (Unitrode easyClean Pt1000 and NH_3_-selective gas membrane electrode) connected to a pH meter (model 713; Methrom, Herisau, Switzerland). The concentration of VFA was analysed using HPLC (System Hitachi Lachrom; Merck, Tokyo, Japan) following the procedure of Ehrlich et al.^55^. Various metabolites were identified and quantified by NMR (Table 2). Samples were processed by filtration via Nanosep 3 k Omega (Pall, Port Washington, NY, USA), with 3 kDa cut-off to remove protein molecules. An amount of 440 μL of sample was mixed with 100 μL of NaHPO_4_ buffer (1 M, pH 7), and 60 μL of 5 mM sodium trimethylsilylpropionate-d_4_ (TSP) (Armar AG, Döttingen, Switzerland) in deuterated water (D_2_O) was used as internal standard. All NMR experiments were performed at 25 °C on a 600 MHz Bruker Avance III HD spectrometer equipped with a Prodigy triple-resonance probe with z-gradient. Quantitative ^1^H spectra were recorded using a 1D-NOESY sequence (τ_mix_ = 10 ms) with presaturation of the water resonance during the relaxation delay. The relaxation delay was 7.5 s and the CW presaturation field strength was set to 30 Hz. The acquisition time was 5 s. The spectral width was 22 ppm centred on the water signal at 4.7 ppm. After 8 dummy scans, 512 scans with 131,072 total data points were accumulated for each spectrum. All spectra were processed with MestReNova14 (Mestrelab Research S. L.). Prior to Fourier transformation the time domain was extended to twice its size by zero-filling and multiplied with an exponential function (LB = 0.15 Hz). The baseline of the resulting spectra was corrected with a polynomial of 3rd order. Metabolites were quantified by comparing their integrals to the integral of the internal standard. The integration method was set to “sum”. For metabolites with more than one ^1^H resonance the following signals were used for quantification: choline (all signals), ethanol (CH_3_), acetaldehyde (HCO), glycerol (CHOH), lactate (CHOH), phenylpropionate (CH_2_COOH), 2-methylbutyrate (CH_3_ at 0.86 ppm, H at 1.39 ppm). Signals were assigned by comparison with data from the Human Metabolome Database at www.hmdb.ca and Bica et al*.*^56^. If necessary, additional data from DQF-COSY, TOCSY, HSQC and HMBC spectra recorded for specific samples were used for this purpose. The total amount of fermentation gas produced was quantified by the water displacement technique as previously described^12^. Fermentation gas samples were then analysed for concentrations of CH_4_ and H_2_ on a gas chromatograph (model 6890 N, Agilent Technologies, Wilmington, DE, USA) equipped with a thermal conductivity detector (to determine H_2_), a flame ionization detector (to determine CH_4_), and a 234 mm × 23 mm column (80/100 mesh, Porapak Q; Fluka Chemie, Buchs, Switzerland).

### DNA extraction

The DNA was extracted in duplicate from 2 mL of incubation liquid using the modified phenol–chloroform bead-beating with QIAquick kit method^57^. The bead beating step was performed for 50 s in a MagNA lyzer (Roche, Basel, Switzerland) with 0.5 mm zirconia/silica bead (Carl Roth, Karlsruhe, Germany). The DNA precipitation step was performed using polyethylene glycol^58^. Quality and quantity of DNA were assessed by NanoDrop One (Witec, Sursee, Switzerland).

### Quantification of relative abundance of microbes by quantitative PCR

The relative abundances of MMC to total archaea and of total archaea to total bacteria were quantified by qPCR using the Roche Lightcycler 96 (Roche, Basel, Switzerland). Each 20 μL reaction consisted of 10 μL SYBR Green I Master Mix (Roche), 1 μL each of 5 μM forward primer and reverse primer, 5 μL of 0.5 ng/μL template and 3 μL nuclease free water. The primers used are described in Supplementary Table S9. The running conditions are described in the Lightcycler 96 manual version 2016 (Roche).

### DNA sequencing and data processing

The microbiome was assessed by metagenomic sequencing. A total of 1089 metagenome assembled genomes (MAGs) were reconstructed. The relative abundance of the prokaryotes was quantified by mOTUs2 profiler^59^ using species level clusters of metagenomic-based Operational Taxonomic Units (mOTUs). The validity of the sequencing pipeline was validated by the Zymo Microbial Community DNA Standard (Supplementary Table S10). The extracted DNA was analysed using the sample library prepared by Illumina Truseq Nano, and sequenced on Novaseq SP 300 cycles Flowcell by Illumina Novaseq 6000 (Illumina, San Diego, CA, USA). Twenty-eight samples were sequenced in the present study generating between 26 and 85 M 150-base-pair paired-end reads per sample. All raw sequences can be accessed through the NCBI at BioProject PRJEB43305, and the code for all the analyses were detailed in submitted publication by Paoli et al*.*^60^.

The sequencing reads from all metagenomes were quality filtered using BBMap^61^ (v.38.71. Available from: https://sourceforge.net/projects/bbmap/). We first removed adapters from the reads, and then removed reads that mapped to quality control sequences (PhiX genome). We discarded low quality reads by applying the parameters trimq = 14, maq = 20, maxns = 0 and minlength = 45. Reads were then merged using bbmerge.sh with a minimum overlap of 16 bases. The merging step results into merged and unmerged reads that are both used from hereon for all analysis steps. Assembly was performed using metaSPAdes^62^ (v3.14) in metagenomic mode. The resulting scaffolded contigs (hereafter scaffolds) were filtered by length (≥ 1000 bp). MAG reconstruction was performed by mapping sequences from all samples against all filtered scaffolds using bwa^63^ (v0.7.17-r1188) with the -a flag and alignments were filtered to be at least 45 bases in length, with an identity ≥ 97% and covering ≥ 80% of the query sequence. Alignment files were processed using the jgi_summarize_bam_contig_depth script to create abundance profiles that were used as input for MetaBAT2^64^ (v2.12.1). Quality of resulting bins were estimated using checkM^65^ (v1.0.13). A total of 1189 bins with a completion ≥ 50% and a contamination < 10% were reported as MAGs and used for downstream analysis. Marker genes from the 1189 MAGs from this study, 410 genomes from the Hungate collection^66^ and 4941 publicly available rumen MAGs^67^ were extracted using fetchMGs (v1.2, available at http://motu-tool.org/fetchMG.html) and 6197 MAGs with ≥ 6 marker genes were used to extend the mOTUs^59^ (v2.5) database. 1154 MAGs were added to existing mOTUs and 5043 MAGs created 2311 new mOTUs (Illustration of workflow given in Supplementary Fig. S2). Next, the 28 Rusitec samples were profiled taxonomically using the mOTUsv2 tool in combination with the extended database using default parameters. Gene calling of the 28 Rusitec assemblies and the 6540 MAGs were called using Prodigal^68^ (v2.6.3) with the parameters -c -q -m -p meta and -c -q -m -p single respectively. Genes were subsequently clustered at 95% identity, keeping the longest sequence as representative using CD-HIT^69^ (v4.8.1) with the parameters-c 0.95 -M 0 -G 0 -aS 0.9 -g 1 -r 0 -d 0. Representative gene sequences were aligned against the KEGG database^70^ (release 2020-02-10) using DIAMOND^71^ (v0.9.30) and filtered to have a minimum query and subject coverage of 70% and requiring a bitScore of at least 50% of the maximum expected bitScore (reference against itself). The MAGs affiliated under mOTUs of interest were collectively analysed as pangenome by OrthoMCL^72^ (v2.0) via Kbase^73^.

### mOTUs cluster capability prediction

Prediction of capability was based on the presence of predicted genes listed in Table S11.

### Statistical evaluation

The statistics program R studio^74^ was used for all evaluations other than multiple comparisons, which was carried out in GraphPad Prism 8.0.0 (GraphPad Software, San Diego, California USA, www.graphpad.com). In preliminary experiment 2, the CH_4_ production was normalized to that of the control (0 ChCl group). Data from the main experiment were subjected to analysis of variance with choline treatment as fixed effect and Rusitec fermenter as experimental unit. Tukey’s method was applied to perform multiple comparisons among treatment means. The mOTUs results were analysed using the vegan package^75^ of R studio, and the Richness and Shannon evenness index was calculated to assess α-diversity. The Mann–Whitney–Wilcoxon Test was performed to establish significant differences in population distribution. Constrained principal coordinate analysis based on Bray Curtis dissimilarity was used to assess β-diversity and dimension reduction. Permutation analysis of variance was used to determine the significance of difference between groups. Differential abundance analyses were carried out by DESeq2^76^. The mOTUs clusters satisfying the statistical cutoff of *P* < 0.05 (Wald-test), the Benjamini–Hochberg false discovery rate adjusted *P* value (*p*adj) of < 0.05 and a log2 fold change of ≥ 2 were considered differentially abundant. Spearman’s Rank correlation coefficient (R_s_) was used for associations of mOTUs with CH_4_ mitigation and H_2_ production. Relation between metabolites and CH_4_ mitigation were established as Pearson Correlation coefficients (r). Cutoffs of *P* < 0.05 and *p*adj < 0.05 were applied to the R_s_ of each mOTUs.

## Supplementary Information

Supplementary Information 1.

Supplementary Information 2.

## Data Availability

All raw sequences are available through the European Nucleotide Archive at BioProject PRJEB43305.

## References

[1] Cubasch U, Stocker TF (2013). Climate Change 2013: the physical science basis. Contribution of Working Group I to the Fifth Assessment Report of the Intergovernmental Panel on Climate Change, Vol 1.

[2] Jackson RB (2020). Increasing anthropogenic methane emissions arise equally from agricultural and fossil fuel sources. Environ. Res. Lett..

[3] Saunois M (2020). The global methane budget 2000–2017. Earth Syst. Sci. Data.

[4] Hobson PN, Stewart CS (1997). The rumen Microbial Ecosystem.

[5] Henderson G (2015). Rumen microbial community composition varies with diet and host, but a core microbiome is found across a wide geographical range. Sci. Rep..

[6] Li Y (2016). The complete genome sequence of the methanogenic archaeon ISO4-H5 provides insights into the methylotrophic lifestyle of a ruminal representative of the Methanomassiliicoccales. Stand. Genom. Sci..

[7] Lang K (2015). New mode of energy metabolism in the seventh order of methanogens as revealed by comparative genome analysis of "Candidatus Methanoplasma termitum". Appl. Environ. Microbiol..

[8] Hoehler T, Losey NA, Gunsalus RP, McInerney MJ, Stams A, Sousa D (2018). Biogenesis of Hydrocarbons.

[9] Neill AR, Grime DW, Dawson RMC (1978). Conversion of choline methyl groups through trimethylamine into methane in the rumen. Biochem. J..

[10] Erdman RA, Sharma BK (1991). Effect of dietary rumen-protected choline in lactating dairy cows. J. Dairy Sci..

[11] Sharma BK, Erdman RA (1989). Effects of dietary and abomasally infused choline on milk production responses of lactating dairy cows. J. Nutr..

[12] Soliva C, Hess H, Makkar HP, Vercoe PE (2007). Measuring Methane Production from Ruminants: Measuring Methane Emission of Ruminants by In Vitro and In Vivo Techniques.

[13] Craciun S, Balskus EP (2012). Microbial conversion of choline to trimethylamine requires a glycyl radical enzyme. PNAS.

[14] Jameson E (2016). Anaerobic choline metabolism in microcompartments promotes growth and swarming of *Proteus mirabilis*. Environ. Microbiol..

[15] Herring TI, Harris TN, Chowdhury C, Mohanty SK, Bobik TA (2018). A bacterial microcompartment is used for choline fermentation by Escherichia coli 536. J. Bacteriol..

[16] EFSA (2011). Scientific Opinion on safety and efficacy of choline chloride as a feed additive for all animal species. EFSA J..

[17] Lewis DJ (1960). Ammonia toxicity in the ruminant. J. Agric. Sci..

[18] Hogan JP (1961). Absorption of ammonia through rumen of sheep. Aust. J. Biol. Sci..

[19] Sprott GD, Patel GB (1986). Ammonia toxicity in pure cultures of methanogenic bacteria. Syst. Appl. Microbiol..

[20] Lewis D (1960). Ammonia toxicity in the ruminant. J. Agric. Sci..

[21] Ungerfeld EM, Rust SR, Burnett R (2007). Increases in microbial nitrogen production and efficiency in vitro with three inhibitors of ruminal methanogenesis. Can. J. Microbiol..

[22] Lundgren BR, Sarwar Z, Pinto A, Ganley JG, Nomura CT (2016). Ethanolamine catabolism in *Pseudomonas aeruginosa* PAO1 is regulated by the enhancer-binding protein EatR (PA4021) and the alternative sigma factor RpoN. J. Bacteriol..

[23] Rychlik JL, LaVera R, Russell JB (2002). Amino acid deamination by ruminal *Megasphaera elsdenii* strains. Curr. Microbiol..

[24] Park K, Lee H (2020). Effects of nitrogen gas flushing in comparison with argon on rumen fermentation characteristics in in vitro studies. J. Anim. Sci. Technol..

[25] Hobson PN, Summers R, Postgate JR, Ware DA (1973). Nitrogen fixation in the rumen of a living sheep. J. Gen. Microbiol..

[26] Harada N, Nishiyama M, Matsumoto S (2001). Inhibition of methanogens increases photo-dependent nitrogenase activities in anoxic paddy soil amended with rice straw. FEMS Microbiol. Ecol..

[27] Haaker H, Klugkist J (1987). The bioenergetics of electron transport to nitrogenase. J FEMS Microbiol. Lett..

[28] Edgren T, Nordlund S (2004). The fixABCX genes in Rhodospirillum rubrum encode a putative membrane complex participating in electron transfer to nitrogenase. J. Bacteriol..

[29] Igai K (2016). Nitrogen fixation and nifH diversity in human gut microbiota. Sci. Rep..

[30] Ungerfeld EM (2015). Shifts in metabolic hydrogen sinks in the methanogenesis-inhibited ruminal fermentation: A meta-analysis. Front. Microbiol..

[31] Leahy SC (2013). The complete genome sequence of *Methanobrevibacter* sp. AbM4. Stand. Genom. Sci..

[32] Hoedt EC (2016). Differences down-under: Alcohol-fueled methanogenesis by archaea present in *Australian macropodids*. ISME J..

[33] Ungerfeld EM, Kohn RA, Sejrsen K, Hvelplund T, Nielsen MO (2006). Ruminant Physiology: Digestion, Metabolism and Impact of Nutrition on Gene Expression, Immunology and Stress.

[34] van Zijderveld SM (2010). Nitrate and sulfate: Effective alternative hydrogen sinks for mitigation of ruminal methane production in sheep. J. Dairy Sci..

[35] Lan W, Yang C (2019). Ruminal methane production: Associated microorganisms and the potential of applying hydrogen-utilizing bacteria for mitigation. Sci. Total Environ..

[36] Loubinoux J, Bronowicki JP, Pereira IA, Mougenel JL, Faou AE (2002). Sulfate-reducing bacteria in human feces and their association with inflammatory bowel diseases. FEMS Microbiol. Ecol..

[37] Gould DH, Cummings BA, Hamar DW (1997). In vivo indicators of pathologic ruminal sulphide production in steers with diet-induced polioencephalomalacia. J. Vet. Diagn. Invest..

[38] Anderson RC, Rasmussen MA, Jensen NS, Allison MJ (2000). Denitrobacterium detoxificans gen. nov., sp. nov., a ruminal bacterium that respires on nitrocompounds. Int. J. Syst. Evol. Microbiol..

[39] Anderson RC (2016). Ruminal fermentation of anti-methanogenic nitrate- and nitro-containing forages in vitro. Front. Vet. Sci..

[40] Zhang ZW (2018). Nitrocompounds as potential methanogenic inhibitors in ruminant animals: A review. Anim. Feed Sci. Tech..

[41] Marounek M, Fliegrova K, Bartos S (1989). Metabolism and some characteristics of ruminal strains of *Megasphaera elsdenii*. Appl. Environ. Microbiol..

[42] Hackmann TJ, Ngugi DK, Firkins JL, Tao J (2017). Genomes of rumen bacteria encode atypical pathways for fermenting hexoses to short-chain fatty acids. Environ. Microbiol..

[43] Janssen PH (2010). Influence of hydrogen on rumen methane formation and fermentation balances through microbial growth kinetics and fermentation thermodynamics. Anim. Feed Sci. Technol..

[44] Greening C (2019). Diverse hydrogen production and consumption pathways influence methane production in ruminants. ISME J..

[45] Gilmour M, Flint HJ, Mitchell WJ (1994). Multiple lactate dehydrogenase activities of the rumen bacterium *Selenomonas ruminantium*. Microbiol..

[46] Chowdhury NP, Kahnt J, Buckel W (2015). Reduction of ferredoxin or oxygen by flavin-based electron bifurcation in *Megasphaera elsdenii*. FEBS J..

[47] Weghoff MC, Bertsch J, Muller V (2015). A novel mode of lactate metabolism in strictly anaerobic bacteria. Environ. Microbiol..

[48] Hernandez J, Benedito JL, Abuelo A, Castillo C (2014). Ruminal acidosis in feedlot: from aetiology to prevention. Sci. World J..

[49] Vuotto C, Barbanti F, Mastrantonio P, Donelli G (2014). *Lactobacillus brevis* CD2 inhibits *Prevotella melaninogenica* biofilm. Oral Dis..

[50] van Lingen HJ (2016). Thermodynamic driving force of hydrogen on rumen microbial metabolism: A theoretical investigation. PLoS One.

[51] Ungerfeld EM, Aedo MF, Martinez ED, Saldivia M (2019). Inhibiting methanogenesis in rumen batch cultures did not increase the recovery of metabolic hydrogen in microbial amino acids. Microorganisms.

[52] Ng F (2016). An adhesin from hydrogen-utilizing rumen methanogen *Methanobrevibacter ruminantium* M1 binds a broad range of hydrogen-producing microorganisms. Environ. Microbiol..

[53] Soliva CR, Amelchanka SL, Duval SM, Kreuzer M (2011). Ruminal methane inhibition potential of various pure compounds in comparison with garlic oil as determined with a rumen simulation technique (Rusitec). Brit. J. Nutr..

[54] Terranova M (2021). Increasing the proportion of hazel leaves in the diet of dairy cows reduced methane yield and excretion of nitrogen in volatile form, but not milk yield. Anim. Feed Sci. Technol..

[55] Ehrlich GG, Goerlitz DF, Bourell JH, Eisen GV, Godsy EM (1981). Liquid chromatographic procedure for fermentation product analysis in the identification of anaerobic bacteria. Appl. Environ. Microbiol..

[56] Bica R (2020). Nuclear magnetic resonance to detect rumen metabolites associated with enteric methane emissions from beef cattle. Sci. Rep..

[57] Henderson G (2013). Effect of DNA extraction methods and sampling techniques on the apparent structure of cow and sheep rumen microbial communities. PLoS One.

[58] Kittelmann S (2013). Simultaneous amplicon sequencing to explore co-occurrence patterns of bacterial, archaeal and eukaryotic microorganisms in rumen microbial communities. PLoS One.

[59] Milanese A (2019). Microbial abundance, activity and population genomic profiling with mOTUs2. Nat. Comm..

[60] Paoli L (2021). Uncharted biosynthetic potential of the ocean microbiome. bioRxiv.

[61] Bushnell, B. BBMap: A fast, accurate, splice-aware aligner. in 9th Annual Genomics of Energy & Environment Meeting. (Lawrence Berkeley National Lab (LBNL), Berkeley, CA, USA). https://www.osti.gov/servlets/purl/1241166 (2014).

[62] Nurk S, Meleshko D, Korobeynikov A, Pevzner PA (2017). metaSPAdes: A new versatile metagenomic assembler. Genome Res..

[63] Li H, Durbin R (2009). Fast and accurate short read alignment with Burrows–Wheeler transform. Bioinformatics.

[64] Kang DD (2019). MetaBAT 2: An adaptive binning algorithm for robust and efficient genome reconstruction from metagenome assemblies. PeerJ.

[65] Parks DH, Imelfort M, Skennerton CT, Hugenholtz P, Tyson GW (2015). CheckM: Assessing the quality of microbial genomes recovered from isolates, single cells, and metagenomes. Genome Res..

[66] Seshadri R (2018). Cultivation and sequencing of rumen microbiome members from the Hungate1000 Collection. Nat. Biotechnol..

[67] Stewart RD (2019). Compendium of 4,941 rumen metagenome-assembled genomes for rumen microbiome biology and enzyme discovery. Nat. Biotechnol..

[68] Hyatt D (2010). Prodigal: Prokaryotic gene recognition and translation initiation site identification. BMC Bioinform..

[69] Fu L, Niu B, Zhu Z, Wu S, Li W (2012). CD-HIT: Accelerated for clustering the next-generation sequencing data. Bioinformatics.

[70] Kanehisa M, Goto S (2000). KEGG: Kyoto encyclopedia of genes and genomes. Nucleic Acid Res..

[71] Buchfink B, Xie C, Huson DH (2015). Fast and sensitive protein alignment using DIAMOND. Nat. Methods.

[72] Li L, Stoeckert CJ, Roos DS (2003). OrthoMCL: Identification of ortholog groups for eukaryotic genomes. Genome Res..

[73] Allen B, Drake M, Harris N, Sullivan T (2017). Using KBase to assemble and annotate prokaryotic genomes. Curr. Protoc. Microbiol..

[74] RStudio Team. R Studio: Integrated development environment for R. Version 1.4.1106 (2021).

[75] Oksanen J (2007). The vegan package. Community Ecol. Pack..

[76] Love MI, Huber W, Anders S (2014). Moderated estimation of fold change and dispersion for RNA-seq data with DESeq2. Genome Biol..

