# Supplementary material for: The rumen microbiome inhibits methane formation through dietary choline supplementation
Source: Sci Rep. 2021 Nov 5;11:21761. doi: 10.1038/s41598-021-01031-w (PMC8571420; doi:10.1038/s41598-021-01031-w)
Supplement: Supplementary file 1 — Supplementary Information 1. [file 41598_2021_1031_MOESM1_ESM.docx]

**Supplementary Information**

**The rumen microbiome inhibits methane formation through dietary choline supplementation**

**Yang Li, Michael Kreuzer, Quentin Clayssen, Marc-Olivier Ebert, Hans-Joachim Ruscheweyh, Shinichi Sunagawa, Carmen Kunz, Graeme Attwood, Sergej Amelchanka, and Melissa Terranova**

**Supplementary information on main experiment**

The fruity smell noted from the choline treatments is speculated to be ethylene. Recently a new enzyme, methylthioalkane reductase encoded by *marKDHB*, was found to be able to produce ethylene from a methionine recycling pathway^1^. The MAGs from the present study harboring *marBDHK* were predicted using BLASTp. All four genes were identified in 16 of 17 genomes of *P. bryantii*, all *Lachnospira multipara/pectinoschiza* (ref_mOTU_v25_03833) genomes and nine of ten genomes of a Lachnospiraceae bacterium (rumen_mOTU_727). This suggests they may be able to produce ethylene which is a known methanogenesis inhibitor^2,3^. However, this requires further validation.

**References**

^1^North, J. A. *et al.* A nitrogenase-like enzyme system catalyzes methionine, ethylene, and methane biogenesis. *Science* **369**, 1094-1098, doi:10.1126/science.abb6310 (2020).

^2^Schink, B. Inhibition of methanogenesis by ethylene and other unsaturated hydrocarbons. *FEMS Microbiol Ecol* **1**, 63-68, doi:10.1111/j.1574-6968.1985.tb01132.x (1985).

^3^Sprott, G. D., Jarrell, K. F., Shaw, K. M. & Knowles, R. Acetylene as an inhibitor of methanogenic bacteria. *J Gen Microbiol* **128**, 2453-2462 (1982).

|  | Day of fermentation | | | | | | | | | | | | | | |
| --- | --- | --- | --- | --- | --- | --- | --- | --- | --- | --- | --- | --- | --- | --- | --- |
| Treatment^1^ | **1** | **2** | **3** | **4** | **5** | **6** | **7** | **8** | **9** | **10** | **11** | **12** | **13** | **14** | **15** |
| Control B | 11.13 | 9.15 | 4.33 | 6.53 | 5.38 | 3.45 | 5.50 | 7.58 | 6.63 | 5.63 | 5.99 | 8.77 | 7.08 | 4.60 | 6.91 |
| Control C | 10.82 | 10.72 | 5.57 | 9.23 | 6.72 | 7.71 | 7.58 | 5.44 | 5.85 | 6.69 | 6.06 | 6.74 | 7.15 | 7.86 | 6.11 |
| Control F | 6.46 | * | 6.50 | 5.19 | 6.60 | 6.56 | 4.88 | 6.44 | 5.95 | 5.53 | 5.14 | 4.74 | 5.15 | 4.41 | 5.63 |
| Control G | 12.75 | 8.99 | 12.74 | 7.33 | 6.51 | 7.07 | 8.18 | 8.10 | 7.63 | 4.84 | 6.67 | 7.05 | 6.26 | 5.46 | 5.82 |
| ChCl B | 16.54 | 9.11 | 4.93 | 7.19 | 7.30 | 7.19 | 1.75 | 1.37 | 0.49 | 0.19 | 0.07 | 0.00 | 0.00 | 0.00 | 0.00 |
| ChCl C | 21.05 | 21.88 | 18.49 | 17.47 | 11.81 | 11.89 | 9.42 | 5.11 | 2.53 | 2.04 | 1.92 | 1.50 | 0.96 | 0.53 | 0.51 |
| ChCl F | * | 13.87 | 11.02 | 7.54 | 5.91 | 4.46 | 2.27 | 1.62 | 0.59 | 0.09 | 0.00 | 0.00 | 0.00 | 0.00 | 0.00 |
| ChCl G | 15.94 | 10.32 | 4.99 | 6.90 | 4.84 | 2.96 | 1.48 | 0.61 | 0.18 | 0.18 | 0.13 | 0.00 | 0.00 | 0.00 | 0.00 |
| ChHCO_3_ B | 18.58 | 14.76 | 12.25 | 10.86 | 9.33 | 7.99 | 4.62 | 1.50 | 0.48 | 0.22 | 0.19 | 0.20 | 0.12 | 0.15 | 0.13 |
| ChHCO_3_ C | * | * | 20.15 | * | 11.52 | 9.86 | 5.60 | 3.81 | 0.80 | 0.17 | 0.27 | 0.25 | 0.17 | 0.19 | 0.24 |
| ChHCO_3_ F | 10.79 | 15.12 | 16.42 | 11.90 | 9.27 | 6.25 | 4.88 | 2.15 | 0.83 | 0.68 | 0.65 | 0.54 | 0.34 | 0.19 | 0.13 |
| ChHCO_3_ G | 13.32 | 11.33 | 9.42 | 6.00 | 2.93 | 2.30 | 1.02 | 0.35 | 0.25 | 0.21 | 0.19 | 0.42 | 0.98 | 1.10 | 0.36 |

**Supplementary Table S1.** Methane production (mmol/d) with zero supplementation (Control) and supplementation of 200 mM choline chloride (ChCl) and 200 mM choline bicarbonate (ChHCO_3_) (data from main experiment). The supplements were dissolved in artificial saliva in order to provide a constant selection pressure. The average artificial saliva flowrate was 403 mL per day. ^1^Groups B and G have been inoculated with rumen fluid from Cow 1, groups C and F have been inoculated with rumen fluid from Cow 2. *Data not available due to gas leak.

|  | Day of fermentation | | | | | | | | | | | | | | |
| --- | --- | --- | --- | --- | --- | --- | --- | --- | --- | --- | --- | --- | --- | --- | --- |
| Treatment^1^ | **1** | **2** | **3** | **4** | **5** | **6** | **7** | **8** | **9** | **10** | **11** | **12** | **13** | **14** | **15** |
| Control B | 0.23 | 0.07 | 0.03 | 0.11 | 0.18 | 0.12 | 0.49 | 0.80 | 0.41 | 0.14 | 0.35 | 0.28 | 0.04 | 0.09 | 0.10 |
| Control C | 0.03 | 0.16 | 0.27 | 0.60 | 0.98 | 0.87 | 1.04 | 0.70 | 0.85 | 0.59 | 0.25 | 0.24 | 0.33 | 0.07 | 0.03 |
| Control F | 0.16 | * | 0.57 | 0.50 | 0.78 | 0.54 | 0.20 | 0.12 | 0.07 | 0.08 | 0.04 | 0.04 | 0.18 | 0.15 | 0.06 |
| Control G | 0.13 | 0.25 | 0.53 | 0.37 | 0.40 | 0.19 | 0.14 | 0.18 | 0.29 | 0.23 | 0.10 | 0.11 | 0.16 | 0.24 | 0.55 |
| ChCl B | 0.25 | 0.23 | 0.25 | 0.14 | 0.19 | 0.30 | 0.12 | 0.25 | 0.31 | 0.56 | 0.30 | 0.56 | 1.09 | 1.33 | 0.59 |
| ChCl C | 0.19 | 0.19 | 1.08 | 1.43 | 1.30 | 1.00 | 1.72 | 1.33 | 1.64 | 2.08 | 1.73 | 0.75 | 0.28 | 0.47 | 1.76 |
| ChCl F | * | 0.50 | 0.53 | 1.19 | 1.40 | 1.79 | 0.88 | 0.77 | 0.59 | 0.52 | 1.56 | 1.00 | 0.94 | 0.93 | 0.95 |
| ChCl G | 0.29 | 0.20 | 0.49 | 0.58 | 0.22 | 0.27 | 0.51 | 0.70 | 1.51 | 2.38 | 1.54 | 1.34 | 1.37 | 1.15 | 1.96 |
| ChHCO_3_ B | 0.05 | 0.07 | 0.11 | 0.14 | 0.06 | 0.04 | 0.28 | 0.58 | 2.23 | 3.16 | 3.81 | 2.56 | 4.39 | 3.82 | 3.39 |
| ChHCO_3_ C | * | * | 0.10 | * | 0.26 | 0.18 | 0.14 | 1.56 | 3.25 | 3.82 | 5.19 | 4.11 | 3.69 | 3.52 | 3.89 |
| ChHCO_3_ F | 0.26 | 0.34 | 0.31 | 0.25 | 0.13 | 0.22 | 0.27 | 0.75 | 2.48 | 2.25 | 2.37 | 2.08 | 2.02 | 2.14 | 3.17 |
| ChHCO_3_ G | 0.03 | 0.15 | 0.63 | 0.74 | 0.58 | 1.74 | 1.83 | 2.18 | 4.90 | 3.04 | 4.24 | 3.51 | 2.90 | 2.92 | 2.02 |

**Supplementary Table S2.** Hydrogen production (mmol/d) with zero supplementation (Control) and supplementation of 200 mM choline chloride (ChCl) and 200 mM choline bicarbonate (ChHCO_3_) (data from main experiment). The supplements were dissolved in artificial saliva in order to provide a constant selection pressure. The average artificial saliva flowrate was 403 mL per day. ^1^Groups B and G have been inoculated with rumen fluid from Cow 1, groups C and F have been inoculated with rumen fluid from Cow 2. *Data not available due to gas leak.

| Target | Oligo name | 5’-3’ | Amplicon size (bp) | Annealing temperature (^o^C) | References |
| --- | --- | --- | --- | --- | --- |
| 16*S* rRNA bacteria | For | CGGYCCAGACTCCTACGGG | 200 | 60 | Lee *et al*. (1996)^1^ |
|  | Rev | TTACCGCGGCTGCTGGCAC |  |  |  |
| 16*S* rRNA MMC | AS1 For | CAGCAGTCGCGAAAACTTC | 485 | 60 | Mihajlovski *et al*. (2010)^2^ |
|  | AS2 Rev | AACAACTTCTCTCCGGCAC |  |  |  |
| 16*S* archaea | 915af | AGGAATTGGCGGGGGAGCAC | 471 | 60 | Watanabe *et al*. (2004)^3^ |
|  | 1386r | GCGGTGTGTGCAAGGAGC |  |  | Skillman *et al*. (2004)^4^ |

**Supplementary Table S9.** Primers used in the present study. ^1^Lee, D. H., Zo, Y. G. & Kim, S. J. Nonradioactive method to study genetic profiles of natural bacterial communities by PCR-single-strand-conformation polymorphism. *Appl Environ Microbiol* **62**, 3112-3120 (1996). ^2^Mihajlovski, A., Dore, J., Levenez, F., Alric, M. & Brugere, J. F. Molecular evaluation of the human gut methanogenic archaeal microbiota reveals an age-associated increase of the diversity. *Environ Microbiol Rep* **2**, 272-280, doi:10.1111/j.1758-2229.2009.00116.x (2010). ^3^Watanabe, T., Asakawa, S., Nakamura, A., Nagaoka, K. & Kimura, M. DGGE method for analyzing 16S rDNA of methanogenic archaeal community in paddy field soil. *FEMS Microbiol Lett* **232**, 153-163, doi:10.1016/S0378-1097(04)00045-X (2004). ^4^Skillman, L. C. *et al.* 16S ribosomal DNA-directed PCR primers for ruminal methanogens and identification of methanogens colonising young lambs. *Anaerobe* **10**, 277-285, doi:10.1016/j.anaerobe.2004.05.003 (2004).

| Taxonomy | mOTU cluster | % abundance estimate |
| --- | --- | --- |
| *Escherichia coli* | ref_mOTU_v25_00095 | 10.0 |
| *Salmonella enterica* | ref_mOTU_v25_00099 | 10.2 |
| *Pseudomonas aeruginosa* | ref_mOTU_v25_00201 | 8.6 |
| *Bacillus subtilis* | ref_mOTU_v25_00278 | 11.1 |
| *Enterococcus faecalis* | ref_mOTU_v25_00318 | 11.1 |
| *Staphylococcus aureus* | ref_mOTU_v25_00340 | 12.0 |
| *Listeria monocytogenes* | ref_mOTU_v25_00712 | 10.9 |
| *Lactobacillus fermentum* | ref_mOTU_v25_01407 | 11.5 |

**Supplementary Table S10.** Validation of relative abundance estimate by comparing expected abundance and abundance estimated by mOTUs2 profiler. ZymoBiomics Microbial Community DNA Standard was sequenced alongside experimental samples. It contains equal 12% abundance of eight bacterial species (expected abundance). The remainder is composed of two fungal species, of which the mOTUs profiler cannot address. The remainder of the % abundance estimate includes the fungal species and the mOTUs that did not meet the minimal marker requirement.

| Function | Genes | Reference |
| --- | --- | --- |
| Choline utilization | *cutC* (choline-trimethylamine lyase) | Craciun *et al*., 2012^1^ |
| Xylan, cellulose degradation | carbohydrate active enzymes listed in database [www.cazy.org](http://www.cazy.org) | Cantarel *et al*., 2009^2^ |
| Xylulose-5-phosphate conversion to acetyl-CoA | xylulose 5 phosphate phosphoketolase | Meile *et al*., 2001^3^ |
| Pyruvate and acetyl-CoA interconversion | pyruvate dehydrogenase | de Kok *et al*., 1998^4^ |
| Pyruvate and lactate interconversion | *ldh* (lactate dehydrogenase) | Garvie 1980^5^ |
| Lactate conversion to propionate | lactoyl-CoA dehydratase, propionate CoA transferase | Hofmeister and Buckel 1992^6^, Selmer *et al*., 2002^7^ |
| Lactate output | possess *ldh* gene but no downstream genes required for conversion to propionate |  |
| Succinate conversion to propionate | methylmalonyl-CoA carboxytransferase, propionyl-CoA carboxylase | Hackmann et al., 2017^8^, Hoffmann *et al*., 1989^9^, Ramos-Vera *et al*., 2011^10^ |
| Butyrate production | butyrate kinase, butyryl-CoA transferase | Hackmann et al., 2017^8^, Oultram *et al*., 1993^11^ |
| Ethanol interconversion with acetyl-CoA, acetaldehyde, and acetate production from acetaldehyde | *adh* (acetaldehyde/alcohol dehydrogenase) | Bosron and Prairie, 1972^12^ |
| Formate production | pyruvate formate-lyase | Knappe *et al*., 1974^13^ |
| Utilization of formate as electron donor | formate hydrogenlyase, *fdh* (formate dehydrogenase) | Finney and Sargent 2019^14^ |
| Ethanolamine utilization | *eutBC* (ethanolamine ammonia lyase) | Thibodeaux and van der Donk 2012^15^ |
| Ethylene production from methionine recycling pathway | *marKDHB* (methylthioalkane reductase) | North *et al*., 2020^16^ |
| Sulfate reduction to hydrogen sulfide | sulfate adenylyltransferase, sulfite reductase | Rabus *et al*., 2013^17^ |
| Nitrate utilisation | nitrate reductase | Moreno-Vivian et al., 1999^18^ |
| Bacteriocin production | *uviB* (bacteriocin), demethyllactenocin mycarosyltransferase, demethyllactenocin carbamoyltransferase, bacitracin export permease protein, penicillin acylase | Collins *et al*., 2017^19^, Russell and Mantovani 2002^20^ |
| Resistance | *marR* (Multiple antibiotic resistance protein), multidrug/daunorubicin/doxorubicin resistance ABC transporter permease and ATP binding protein, streptothricin hydrolase, phosphinothricin *N*-acetyltransferase, arsenical-resistance protein, penicillin-binding protein, methicillin resistance mecR1 protein, β-lactamase, tunicamycin resistance protein, vancomycin resistance protein, bicyclomycin resistance protein, tetracycline resistance protein | Russell and Houlihan 2003^21^, Sabino *et al*., 2019^22^ |

**Supplementary Table S11.** Metabolic capability prediction. ^1^Craciun, S. & Balskus, E. P. Microbial conversion of choline to trimethylamine requires a glycyl radical enzyme. *PNAS USA* **109**, 21307-21312, doi:10.1073/pnas.1215689109 (2012). ^2^Cantarel, B. L. *et al.* The Carbohydrate-Active EnZymes database (CAZy): an expert resource for Glycogenomics. *Nucleic Acids Res* **37**, D233-238, doi:10.1093/nar/gkn663 (2009). ^3^Meile, L., Rohr, L. M., Geissmann, T. A., Herensperger, M. & Teuber, M. Characterization of the D-xylulose 5-phosphate/D-fructose 6-phosphate phosphoketolase gene (*xfp*) from *Bifidobacterium lactis*. *J Bacteriol* **183**, 2929-2936, doi:10.1128/JB.183.9.2929-2936.2001 (2001). ^4^de Kok, A., Hengeveld, A. F., Martin, A. & Westphal, A. H. The pyruvate dehydrogenase multi-enzyme complex from Gram-negative bacteria. *Biochim Biophys Acta* **1385**, 353-366, doi:10.1016/s0167-4838(98)00079-x (1998). ^5^ Garvie, E. I. Bacterial lactate-dehydrogenases. *Microbiol Rev.* **44**, 106-139, doi:Doi 10.1128/Mmbr.44.1.106-139.1980 (1980). ^6^ Hofmeister, A. E. & Buckel, W. (R)-lactyl-CoA dehydratase from *Clostridium propionicum*. Stereochemistry of the dehydration of (R)-2-hydroxybutyryl-CoA to crotonyl-CoA. *FEBS* **206**, 547-552, doi:10.1111/j.1432-1033.1992.tb16958.x (1992). ^7^Selmer, T., Willanzheimer, A. & Hetzel, M. Propionate CoA-transferase from *Clostridium propionicum*. Cloning of gene and identification of glutamate 324 at the active site. *FEBS* **269**, 372-380, doi:10.1046/j.0014-2956.2001.02659.x (2002). ^8^Hackmann, T. J., Ngugi, D. K., Firkins, J. L. & Tao, J. Genomes of rumen bacteria encode atypical pathways for fermenting hexoses to short-chain fatty acids. *Environ Microbiol* **19**, 4670-4683, doi:10.1111/1462-2920.13929 (2017). ^9^Hoffmann, A., Hilpert, W. & Dimroth, P. The carboxyltransferase activity of the sodium-ion-translocating methylmalonyl-CoA decarboxylase of *Veillonella alcalescens*. *FEBS* **179**, 645-650, doi:10.1111/j.1432-1033.1989.tb14596.x (1989). ^10^Ramos-Vera, W. H., Weiss, M., Strittmatter, E., Kockelkorn, D. & Fuchs, G. Identification of missing genes and enzymes for autotrophic carbon fixation in crenarchaeota. *J Bacteriol* **193**, 1201-1211, doi:10.1128/JB.01156-10 (2011). ^11^Oultram, J. D., Burr, I. D., Elmore, M. J. & Minton, N. P. Cloning and sequence analysis of the genes encoding phosphotransbutyrylase and butyrate kinase from *Clostridium acetobutylicum* NCIMB 8052. *Gene* **131**, 107-112, doi:10.1016/0378-1119(93)90677-u (1993). ^12^Bosron, W. F. & Prairie, R. L. Triphosphopyridine nucleotide-linked aldehyde reductase .1. Purification and properties of enzyme from pig kidney-cortex. *J Biol Chem* **247**, 4480 (1972). ^13^Knappe , J., Blaschkowski, H. P., Grobner, P. & Schmitt, T. Pyruvate formate-lyase of *Escherichia coli*: the acetyl-enzyme intermediate. *FEBS* **50**, 253-263, doi:10.1111/j.1432-1033.1974.tb03894.x (1974). ^14^Finney A. J. & Sargent F. in *Advances in Microbial Physiology* *Vol. 74:* *Chapter Eight - Formate hydrogenlyase: A group 4 [NiFe]-hydrogenase in tandem with a formate dehydrogenase.* (ed Poole R. K.) 465-486 (Academic Press, 2019). ^15^Thibodeaux, C. J. & van der Donk, W. A. Converging on a mechanism for choline degradation. *PNAS USA* **109**, 21184-21185, doi:10.1073/pnas.1219534110 (2012). ^16^North, J. A. *et al.* A nitrogenase-like enzyme system catalyzes methionine, ethylene, and methane biogenesis. *Science* **369**, 1094-1098, doi:10.1126/science.abb6310 (2020). ^17^Rabus, R., Hansen, T. A. & Widdel, F. in *The Prokaryotes: Prokaryotic physiology and biochemistry*: *Dissimilatory sulfate- and sulfur-reducing prokaryotes* (eds Eugene Rosenberg *et al.*) 309-404 (Springer Berlin Heidelberg, 2013) ^18^Moreno-Vivian, C., Cabello, P., Martinez-Luque, M., Blasco, R. & Castillo, F. Prokaryotic nitrate reduction: molecular properties and functional distinction among bacterial nitrate reductases. *J Bacteriol* **181**, 6573-6584, doi:10.1128/JB.181.21.6573-6584.1999 (1999). ^19^Collins, F. W. J. *et al.* Bacteriocin gene-trait matching across the complete *Lactobacillus* pan-genome. *Sci Rep* **7**, 3481, doi:10.1038/s41598-017-03339-y (2017). ^20^Russell, J. B. & Mantovani, H. C. The bacteriocins of ruminal bacteria and their potential as an alternative to antibiotics. *J Mol Microbiol Biotechnol* **4**, 347-355 (2002). ^21^Russell, J. B. & Houlihan, A. J. Ionophore resistance of ruminal bacteria and its potential impact on human health. *FEMS Microbiol Rev* **27**, 65-74, doi:10.1016/S0168-6445(03)00019-6 (2003). ^22^Sabino, Y. N. V. *et al.* Characterization of antibiotic resistance genes in the species of the rumen microbiota. *Nat Comm* **10**, 5252, doi:10.1038/s41467-019-13118-0 (2019).

**
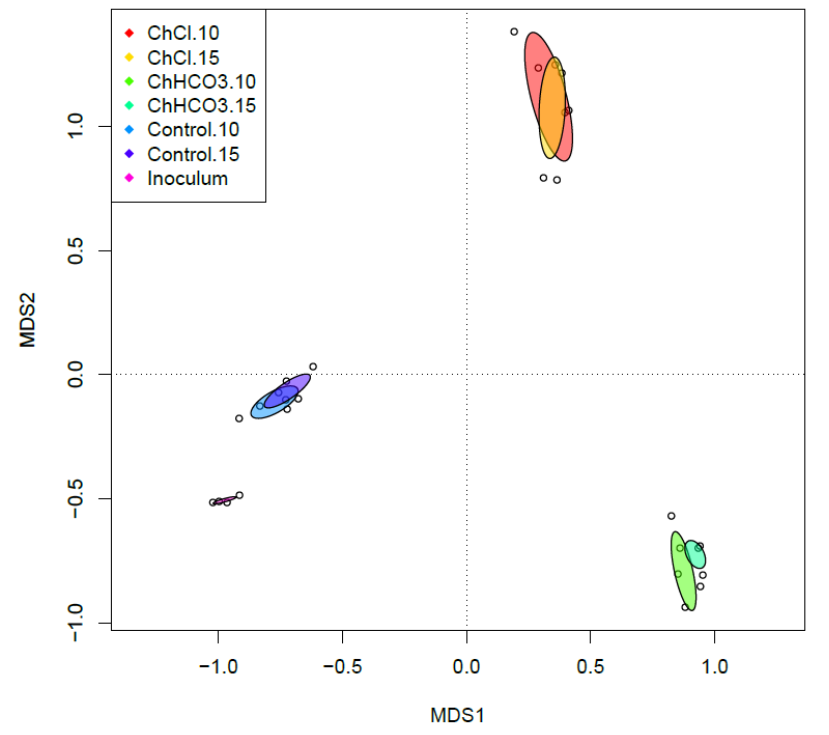
**

**Supplementary Figure S1.** β-diversity as assessed by Constrained Principal Coordinates Analysis based on Bray Curtis dissimilarity of relative prokaryote abundance of inoculum, control, choline chloride (ChCl) and choline bicarbonate (ChHCO_3_) (day 10 (.10) and 15 (.15) displayed by ordiplot) (data from main experiment).

Inoculum

Control

Choline bicarbonate

Choline chloride

C1

C2

C1

C2

**C1**

**C2**

C1

C2

C1

C2

**
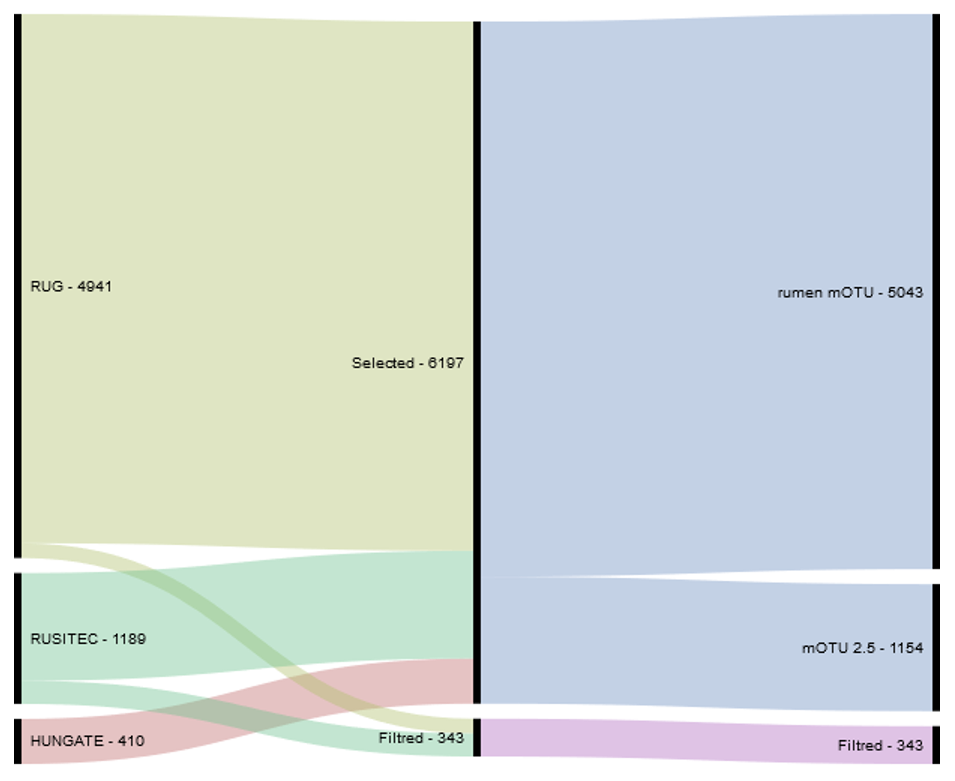
**

**Supplementary Figure S2.** Alluvial graph depicting the workflow of processing metagenome assembled genomes (MAGs) to reference metagenomic-based Operational Taxonomic Units (mOTUs). A total of 1189 MAGs were reconstructed from the current experiment, alongside 4941 MAGs from rumen uncultured genomes (RUGs) by Stewart *et al.*, and 410 available genomes from Hungate collection by Seshadri et al. 6197 genomes with 6 or more mOTUs marker genes are selected to form 2311 new rumen mOTUs from 5043 MAGs. 1154 MAGs were added to existing mOTUs (data from main experiment).
